# A Miniaturized QuEChERS Method Combined with Ultrahigh Liquid Chromatography Coupled to Tandem Mass Spectrometry for the Analysis of Pyrrolizidine Alkaloids in Oregano Samples

**DOI:** 10.3390/foods9091319

**Published:** 2020-09-18

**Authors:** Sergio Izcara, Natalia Casado, Sonia Morante-Zarcero, Isabel Sierra

**Affiliations:** Departamento de Tecnología Química y Ambiental, E.S.C.E.T, Universidad Rey Juan Carlos, C/Tulipán s/n, Móstoles, 28933 Madrid, Spain; sergio.izcara@urjc.es (S.I.); natalia.casado@urjc.es (N.C.); sonia.morante@urjc.es (S.M.-Z.)

**Keywords:** pyrrolizidine alkaloids, natural toxins, aromatic herbs, oregano, miniaturization, UHPLC-MS/MS, µ-QuEChERS, food safety

## Abstract

Recent and unexpected food alerts about relatively high amounts of pyrrolizidine alkaloids in oregano samples have stressed the need to develop analytical strategies to ensure food safety in this type of foodstuff. Accordingly, this work presents the development of a miniaturized strategy based on the QuEChERS (quick, easy, cheap, effective, rugged and safe) method combined with ultrahigh liquid chromatography coupled to tandem mass spectrometry (UHPLC-MS/MS) for the determination of 21 pyrrolizidine alkaloids suggested by the European Food Safety Authority to be monitored in food. The analytical method was properly validated, with overall average recoveries from 77 to 96% and relative standard deviations <13% (*n* = 9). The method proved to be a sustainable analytical strategy which meets green analytical chemistry principles as it showed good performance by using small amounts of sample (0.2 g), organic solvents (1000 µL), clean-up sorbents (175 mg) and partitioning salts (0.65 g). Its feasibility was verified through the analysis of 23 oregano samples. Of the samples analyzed, 100% were contaminated, with an average concentration of 1254 µg/kg. Lasiocarpine, lasiocarpine *N*-oxide, europine, europine *N*-oxide, senecivernine, senecionine, echimidine *N*-oxide, lycopsamine *N*-oxide and intermedine *N*-oxide were the alkaloids which significantly contributed to the contamination of the samples.

## 1. Introduction

In the last two years (2019–2020), the significant increase in the number of food alerts reported on the Food and Feed Safety Alerts (RASFF) portal related to the presence of pyrrolizidine alkaloids (PAs) and their oxidized forms (pyrrolizidine alkaloids *N*-oxides, PANOs) in different food products has been striking [1]. In these two years, these alerts have been raised for different types of teas, food supplements, pollen, spices and aromatic herbs. Nonetheless, surprisingly, most of them have been reported in oregano (19 out of 38), Germany being the country with the highest number of cases detected and alerts issued, revealing unexpected and concerningly high levels of these contaminants in this aromatic herb (among 6660 and 133,870 µg/kg) (Table S1) [1]. Consequently, this has led to the worldwide withdrawal of many oregano products from the market in recent years.

PAs are natural toxins produced by the secondary metabolism of plants as a defense mechanism against herbivores and insects. To date, more than 600 different types of PAs and their PANOs have been identified in a wide variety of plant species (>6000), but the great majority of them (about 95%) belong to the families of Asteraceae, Fabaceae, Boraginaceae, Orchidaceae and Apocynaceae [2]. Nevertheless, the major sources of PAs consumption in humans seem to be products contaminated with these PAs-producing plants. Not all PAs and PANOs are toxic, but those with an unsaturation in 1,2 position and at least one ester bond, display hepatotoxicity, as they act as prototoxins which can be activated in the liver by cytochrome P450 into reactive pyrrole intermediates, which can lead to cellular adducts [2,3]. The human intake of these compounds is mainly associated with liver damage, causing hepatic veno-occlusive disease (HVOD), which may lead to liver cirrhosis and liver failure. Moreover, it may also lead to pulmonary hypertension, cardiac hypertrophy, degenerative kidney injuries or even death [3,4]. Indeed, some PAs are considered genotoxic and carcinogenic compounds and have been classified as “possibly carcinogenic to humans” (category 2B) by the International Agency for Research on Cancer (IARC) [3]. Thus, their occurrence in food should be considered a relevant food safety issue.

Due to their potential risk for human health, between 2007 and 2017, the European Food Safety Authority (EFSA) addressed different scientific opinions about the increasing concern of the presence of PAs in food [2,5,6,7,8]. However, these reports concluded that the exposure levels of the population to PAs are still uncertain. Consequently, to date, maximum concentration levels of these compounds in food have not yet been regulated because of this lack of data. Thus, further investigation is required, being necessary to develop sensitive analytical methods enabling the accurate identification and quantification of these contaminants at very low concentration levels in a wide range of products to ensure their food safety. For this purpose, the EFSA has recommended a set of 17 PAs/PANOs to be monitored in food items (intermedine, lycopsamine, intermedine *N*-oxide, lycopsamine *N*-oxide, senecionine, senecivernine, senecionine *N*-oxide, senecivernine *N*-oxide, seneciphylline, seneciphylline *N*-oxide, retrorsine, retrorsine *N*-oxide, echimidine, echimidine *N*-oxide, lasiocarpine, lasiocarpine *N*-oxide and senkirkine) [8]. Nonetheless, increasing the number of PAs/PANOs monitored in food from 17 to 21 is currently being considered, including europine and heliotrine, as well as their respective *N*-oxides, because of their notable occurrence in some foods [9].

Nevertheless, despite the relatively high amounts of PAs recently identified in oregano, to the best of our knowledge, current works focusing on the detection of PAs and their PANOs in aromatic herbs and spices are very scarce in the literature [9,10,11,12], as research has mainly focused on other types of products such as honey, teas and food supplements [13,14,15,16,17,18,19,20,21]. In these few works, it is suggested that aromatic herbs and spices can be widely contaminated with PAs-producing plants during coharvesting or in an intentional way by adulteration. Indeed, it has been revealed that oregano is one of the most extensively adulterated aromatic herbs [22], which may explain the high occurrence of PAs/PANOs in it. With the adulteration of oregano with other herbs not declared, producers would be gaining an economic benefit as they would be selling less raw material as if it were 100% oregano. Thus, they would be committing food fraud. Therefore, it is of high interest to extensively study this matrix.

On the other hand, nowadays, the current trend within the analytical chemistry field is to move towards the development of “greener” methodologies by scaling down conventional analytical operations and miniaturizing the extraction procedures [23,24]. This involves minimal consumption of solvents and samples, as well as fewer sample treatment steps and the reduction in hazardous reagents and wastes. Therefore, this leads to the development of quicker, cheaper, more cost-effective and more environmentally friendly extraction procedures which enable green analytical chemistry (GAC) requirements to be met [25,26]. Indeed, to the best of our knowledge, the determination of PAs/PANOs under microextraction conditions has not been reported in previous works [26].

Therefore, the aim of this work was to develop a sustainable analytical methodology using ultrahigh liquid chromatography coupled to tandem mass spectrometry (UHPLC-MS/MS) to monitor the presence of the 21 PAs/PANOs suggested by the EFSA in oregano samples in order to broaden knowledge about the occurrence of these contaminants in aromatic herbs and ensure their food safety. Accordingly, the multicomponent extraction of the target analytes from the matrix was achieved by the miniaturization of the QuEChERS (quick, easy, cheap, effective, rugged and safe) procedure by reducing the amount of sample, solvent, salts and adsorbents employed, leading to an improved cost-effective and environmentally friendly microextraction method, which meets the GAC principles.

## 2. Materials and Methods

### 2.1. Chemicals, Reagents and Standard Solutions

Methanol (MeOH) and acetonitrile (ACN) LC-MS grade, dimethyl sulfoxide (DMSO), anhydrous magnesium sulphate (MgSO_4_), sodium chloride (NaCl), sodium citrate tribasic dehydrate, sodium citrate dibasic sesquihydrate and primary-secondary amine (PSA) sorbent were purchased from Scharlab (Barcelona, Spain). Formic acid and ammonium acetate LC-MS grade were supplied by Fluka (Busch, Switzerland). Water (resistivity 18.2 MΩ cm) was obtained from a Millipore Milli-Q-System (Billerica, MA, USA). Standards of the target PAs and related PANOs were all high purity grade (≥90%) and were acquired from PhytoLab GmbH & Co. KG (Vestenbergsgreuth, Germany), except retrorsine, which was from Sigma-Aldrich (St. Louis, MO, USA). Individual solutions of each compound (1000 μg/mL) were prepared in MeOH, except for retrorsine, intermedine, lycopsamine, senecionine, seneciphylline, heliotrine, heliotrine *N*-oxide, europine and europine *N*-oxide, which were prepared in ACN/DMSO (4/1, *v/v*) due to their solubility. A multicomponent standard solution (1 μg/mL) containing a mixture of the 21 compounds was prepared in water. Working standard solutions at different concentration levels were prepared by appropriate dilution of the multicomponent standard solution with water to develop and optimize the analytical procedure. All solutions were stored at −20 °C.

### 2.2. Samples and Extraction Procedure

Dry oregano samples from different geographical origins and with different types of farming (conventional and organic) were purchased from different local supermarkets in Madrid (Spain). An oregano sample on the branch was also collected from a wild crop field in Toledo (Spain). The samples were codified by indicating in the first letter their type of farming as follows: W for wild, O for organic and C for conventional farming. Additionally, the code for samples belonging to the same trademark but with a different lot number was a final A or B letter (for sample details, see Supplementary Materials Table S2). All samples were separately milled to a fine powder for their homogenization and stored until their analysis.

The sample extraction procedure was based on a miniaturization of the QuEChERS procedure proposed by Anastassiades et al. (2003) [27], with the addition of citrate buffer to keep the pH constant during extraction (pH 5.5) [28], by means of significantly reducing the original amounts of sample, organic solvents, partitioning salts and adsorbents employed. Accordingly, the μ-QuEChERS procedure proposed in this work was as follows: about 0.2 g of sample, weighed using an analytical balance with resolution equal to 0.1 mg, were mixed with 1 mL water, vortexed for 1 min and incubated under stirring for 30 min to allow the dry matrix to absorb the solvent. Subsequently, 1 mL ACN was added. The mixture was vortexed for 1 min and stirred for 30 min. Then, 0.65 g of the partitioning salts mixture (MgSO_4_, NaCl, sodium citrate tribasic dehydrate and sodium citrate dibasic sesquihydrate in proportion 4:1:1:0.5) was added. The mixture was vortexed for 1 min, followed by ultrasound agitation for 5 min and centrifuged 10 min at 6000 rpm. An aliquot from the upper part of the extract corresponding to the ACN fraction was transferred into an Eppendorf containing 150 mg of MgSO_4_ and 25 mg of PSA. The mixture was vortexed for 1 min and centrifuged for 5 min at 10,000 rpm. Then, the supernatant of the purified extract was filtered through a 0.45 μm PTFE filter membrane and injected in the UHPLC-MS/MS system.

### 2.3. UHPLC-MS/MS Analysis

**Figure 1:**
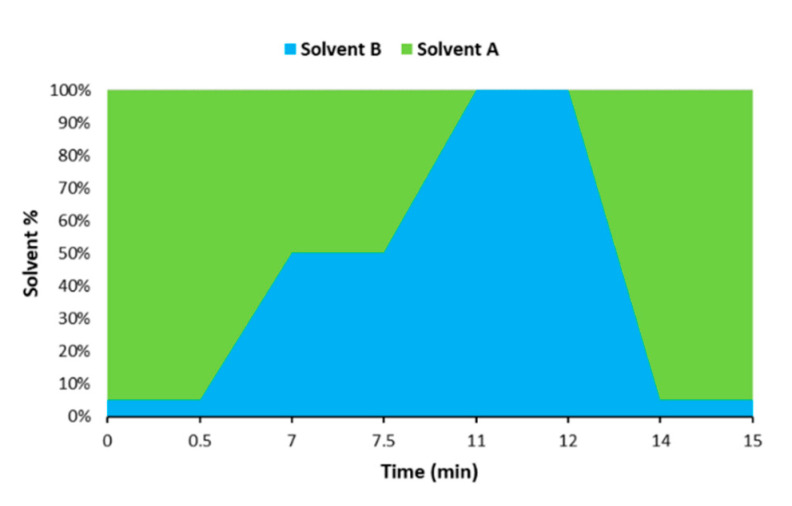
Graphical representation of the gradient used for the chromatographic analysis.

The chromatographic separation was achieved with an UHPLC system (Dionex UltiMate 3000, Thermo Scientific, Waltham, MA, USA) coupled to an ion-trap tandem mass spectrometer detector (Bruker) and a Luna Omega Polar C18 column (100 mm × 2.1 mm, 1.6 μm particle size, Phenomenex, Torrance, CA, USA) at 25 °C. A gradient elution was carried out by combining solvent A (water containing 0.2% formic acid and 5 mM ammonium acetate) and solvent B (MeOH containing 10 mM ammonium acetate) as follows: 5% B (0–0.5 min), 5–50% B (0.5–7 min), 50% B (7–7.5 min), 50–100% B (7.5–11 min), 100% B (11–12 min), 100–5% B (12–14 min). The system was re-equilibrated with the initial composition for 1 min prior to next injection, yielding a total analysis time of 15 min (Figure 1).

The flow rate was 0.250 mL/min, and the injection volume 2 μL. Mass spectrometry (MS) acquisition was achieved with electrospray ionization interface (ESI) operating in positive ion mode. Capillary voltage was set at −4500 V and the end plate offset at −500 V. The nebulizer was held at 20 psi, the dry gas at 10 L/min and the dry temperature at 200 °C. ESI source parameters were optimized by direct infusion of each analyte in pure standard solutions (5 μg/mL) at a flow rate of 4 μL/min. To achieve the maximum total ion current (TIC) signal, different parameters were manually optimized in positive mode within the mass range of 70–700 m/z. Table 1 lists the retention time, mass spectrum parameters and product ions of the target analytes under the conditions described.

### 2.4. Statistical Analysis

Oregano samples were analyzed in triplicate. The data were subjected to one-way analysis of variance (ANOVA) and a Duncan multiple range test, considering significant differences at *p ≤* 0.05. SPSS 19.0 software was used for the statistical analysis.

## 3. Results and Discussion

### 3.1. Optimization of the Chromatographic Separation

One of the major issues in the individual analysis of PAs and PANOs is the co-occurrence of isomers, as they have the same molecular weight and cannot be distinguished by MS, so it is not always possible to achieve their baseline chromatographic separation because they coelute. Among the PAs/PANOs recommended by the EFSA to be monitored in food, this is the case for intermedine/lycopsamine and senecionine/senecivernine, as well as their *N*-oxide derivatives. Moreover, according to the literature, the separation of intermedine/lycopsamine and their *N*-oxides can be achieved under acidic chromatographic conditions, whereas, in contrast, the separation of senecionine/senecivernine and their *N*-oxides is achieved under basic conditions [20,29]. For this reason, many authors instead of including these 8 isomers only analyze some of them excluding the other ones [19,29,30] or perform separately acid and basic methods [20]. In fact, to date, as far as we know, there is only one method recently published in the literature which describes the complete chromatographic separation of all the PAs/PANOs recommended by the EFSA including the separation of the isomers [13]. This was achieved with a Synergi™ Polar-RP C18 chromatographic column (150 mm × 2.0 mm, 4 μm particle size, Phenomenex, Torrance, CA, USA) employing a mobile phase gradient with water and ACN/water (95/5, *v/v*) as solvents A and B, both containing formic acid and ammonium formate. Analytes were separated within 16.5 min, yielding a total run time of 23 min. Nevertheless, under these conditions, some of the isomers coeluted with other compounds which are not included in the EFSA recommendations, such as indicine with lycopsamine and intermedine *N*-oxide with indicine *N*-oxide. Therefore, they were reported as a combined group [13].

Accordingly, in order to achieve the separation of the 21 PAs/PANOs recommended by the EFSA, in this work, two different columns were tested under the same conditions at 30 °C: ACE Excel 2 C18-PFP column (100 mm × 2.1 mm, 2 μm particle size; ACE, Aberdeen, UK) and Luna Omega Polar C18 column (column specifications in Section 2.3). The ACE Excel 2 C18-PFP column combines a C18 ligand with pentafluorophenyl (PFP) groups to increase selectivity, and it is recommended by the manufacturer for the separation of halogenated aromatic compounds, regioisomers and analytes with differing shape constraints. Thus, it could be suitable to achieve the separation of PAs/PANOs isomers. Nevertheless, the authors which used a similar stationary phase with PFP to achieve the separation of different PAs and PANOs did not include in their methods all the isomers recommended by the EFSA [31,32]. On the other hand, the Luna Omega Polar C18 column provides an equilibrate separation of both polar and hydrophobic compounds with the same efficiency, which may be suitable as PAs and PANOs present different polarity among them. As far as we know, this type of column has never been used before for the separation of these compounds.

Both columns were evaluated using acidic chromatography with different organic solvents. In this sense, water was combined with ACN, MeOH or ACN/MeOH (1/1, *v/v*) as the mobile phase, both containing 0.1% of formic acid. First, a linear gradient elution was used, which started with a high aqueous content (95% water), and gradually the organic solvent increased to 100% in 14 min. Although, in general, authors have used ACN rather than MeOH as the organic phase to achieve the chromatographic separation of PAs and PANOs [13,17,18,19,29], it was observed that with ACN in both columns, the majority of the compounds eluted in the first 1–2 min, leading to coelution of those compounds with similar masses. On the other hand, with the mixture ACN/MeOH (1/1, *v/v*), the separation improved, but the isomers coeluted. Conversely, although retention times were longer with MeOH, better separation of the analytes was achieved, enabling the separation of intermedine/lycopsamine and their *N*-oxides. Moreover, MeOH is a cheaper solvent than ACN. In all cases, better separation efficiency was achieved with the Luna Omega Polar C18 column, which additionally enabled, with MeOH, partial separation of senecivernine/senecionine *N*-oxides, probably due to its smaller particle size. Thus, this column and MeOH were selected for further analysis.

Subsequently, to optimize the chromatographic separation, ammonium acetate (5 mM) was tested as additive in the mobile phase alone and in combination with formic acid (0.1%). Additionally, the elution gradient was modified to make it slower to improve separation of senecivernine/senecionine isomers. In this sense, the gradient started with a high aqueous content (95% water) and decreased to 50% in the first 7 min, an isocratic step was included for 2 min and then decreased to 0% in 2 min and was kept for 2 more min, returning to initial conditions in the next 2 min. With the new gradient, the separation efficiency improved, and it was observed that using only 5 mM ammonium acetate as additive without formic acid lead to an inversion of the elution order. The PANOs eluted before their corresponding PAs, unlike in acidic chromatography where PAs eluted before their PANOs. This effect has also been reported by other authors [9]. Nevertheless, when using only ammonium acetate as additive, all isomers coeluted.

**Figure 2:**
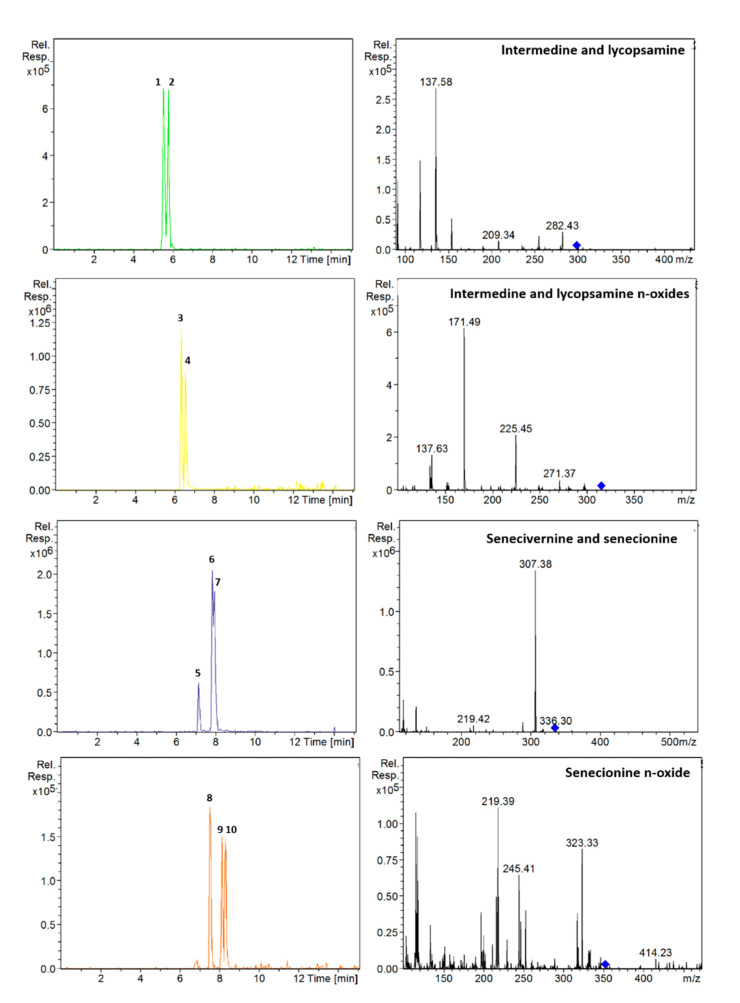
Extracted ion chromatograms and mass spectra (MS^2^) of: intermedine (1), lycopsamine (2), intermedine *N*-oxide (3), lycopsamine *N*-oxide (4), seneciphylline (5), senecivernine (6), senecionine (7), seneciphylline *N*-oxide (8), senecivernine *N*-oxide (9) and senecionine *N*-oxide (10) in a standard solution containing 100 µg/L of each analyte. Chromatographic conditions as Table 1.

On the other hand, it was observed that the mixture of ammonium acetate with formic acid in the aqueous phase combined with the addition of ammonium acetate in the organic phase improved the shape and intensity of the peaks. Therefore, the effect of different concentrations of formic acid (0.1, 0.2 and 0.5%) and ammonium acetate (5, 10 and 20 mM) in the different phases was investigated. The best results were achieved using 0.2% formic acid and 5 mM ammonium acetate in the aqueous phase, and MeOH with 10 mM ammonium acetate. Under these conditions, peaks were narrower and the separation of intermedine/lycopsamine isomers improved. Finally, different temperatures were tested (20, 25, 30, 45 °C), the most suitable being 25 °C. Since senecivernine/senecionine and their *N*-oxides eluted in the isocratic step of the elution gradient, the flow decreased at this point to 0.15 mL/min to try to improve their separation. However, it was not effective. Conversely, reducing the isocratic step in time from 7 to 7.5 min, improved the separation of the senecivernine/senecionine *N*-oxides, and partial separation was achieved for senecivernine/senecione (Figure 2). Thus, under the final conditions, the 21 PAs/PANOs recommended by the EFSA were separated within 10.5 min, yielding a total run time of 15 min. This means a reduction of 8 min compared to the method described above [13]. Retention times are listed in Table 1, and chromatograms of each analyte with their corresponding mass spectra are provided in Figure S1 of the Supplementary Materials.

### 3.2. Extraction Procedure

**Figure 3:**
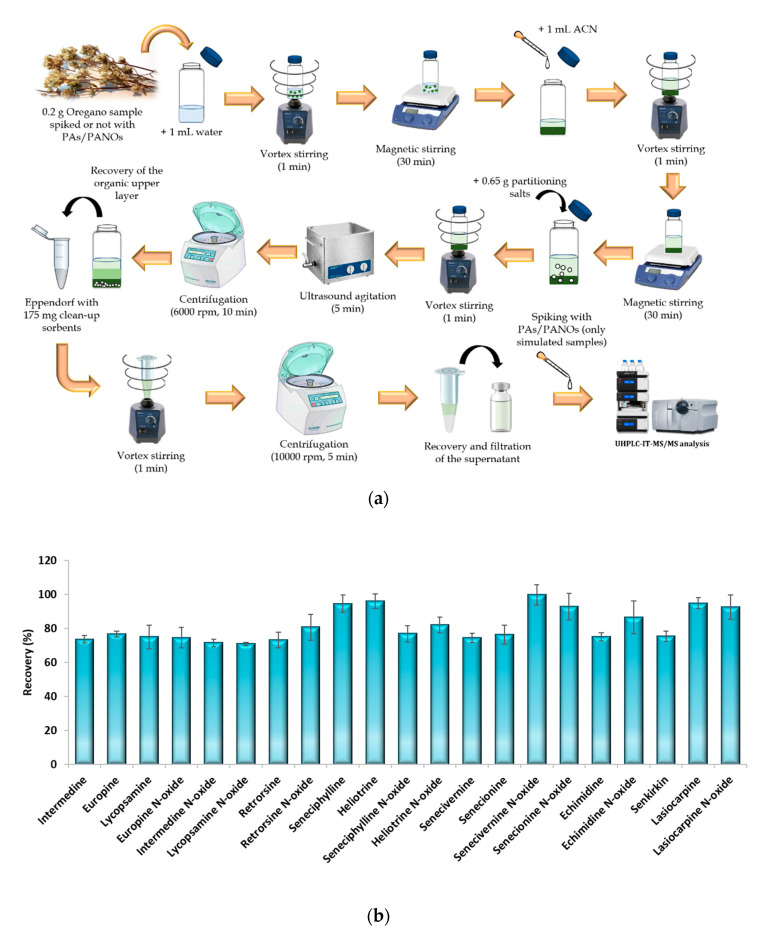
(**a**) Schematic representation of the µ-QuEChERS extraction procedure and (**b**) recovery percentages obtained from the analysis of three spiked replicates of an oregano sample (100 μg/kg of each analyte) extracted by the µ-QuEChERS procedure. Error bars represent the standard deviation of sample replicates (*n* = 3).

The μ-QuEChERS procedure was based on the original method proposed by Anastassiades et al. (2003) [27] with the addition of citrate buffer to preserve pH during extraction [28]. The conventional QuEChERS method is an extraction and clean-up technique based on dispersion which was originally created as an environmentally friendly and cheap procedure to achieve the multiresidue extraction of more than 200 pesticides from fruit and vegetable samples [27]. The original method involves using large amounts of sample (10 g), organic solvents (10 mL) and partitioning salts (5 or 6.5 g) [27,28]. Nevertheless, since its origin as a sample preparation method, the QuEChERS concept has evolved and spread to be adapted to other analytes and matrices. In this sense, for samples containing less than 25% water, such as cereals, dried fruits, honey and spices, the initial amount of sample has to be reduced to 1–5 g, and they require the addition of 10 mL water before their extraction [33]. Particularly, for spices, the sample amount used must be 2 g [33]. Therefore, in order to achieve the miniaturization of the QuEChERS procedure, the amounts used of sample, solvents and partitioning salts were significantly reduced by ten times according to the original QuEChERS procedure. For the dispersive solid-phase extraction step, 175 mg clean-up sorbents (25 mg PSA and 150 mg MgSO_4_) per mL of extract are recommended [33]. Therefore, as the miniaturized procedure led to 1 mL of organic sample extract, these amounts of sorbents were used in the clean-up step. Figure 3a shows the scheme of the μ-QuEChERS procedure proposed.

To check the efficiency of the miniaturized procedure, a recovery assay was carried out. For this purpose, five replicates of a sample (C-S15-A, as it presented the lowest values of PAs/PANOs) were subjected simultaneously to the extraction procedure in the following way: three replicates of the sample were spiked with 0.2 mL of a working standard solution of 100 µg/L containing all the target analytes (100 µg/kg of each analyte referred to the sample weighted), allowing a 20 min period for equilibration prior to the sample extraction procedure; another replicate of the sample (denoted as a simulated sample) was extracted in the same way as the others, but it was spiked with the known-amount of the analytes at the end of the sample treatment procedure (Figure 3a); the last replicate sample was extracted without spiking, so it was considered a blank sample. To calculate the recovery values, the areas of the spiked samples were compared with the areas obtained for the simulated sample, while the blank samples were used to quantify the target analytes in the samples analyzed. Good recovery results were achieved with this miniaturized procedure, as satisfactory recovery values were achieved for all the target compounds (Figure 3b). This suggests that the QuEChERS procedure can be successfully scaled down and be effective enough without a high consumption of solvents and reagents, leading to a cost-effective and environmentally friendly improved strategy.

### 3.3. Method Validation and Analysis of Samples

The proposed methodology was properly validated in terms of linearity, matrix effects, limits of detection and quantification, accuracy and precision. Since there is no official regulation for the validation of analytical methods to monitor the presence of PAs and PANOs in food or feed products, the validation procedures guidelines established in the SANTE/11813/2017 document for the analytical quality control of pesticide residues in food and feed [34] and the criteria established in the regulation EC No 401/2006 [35] were followed to validate the proposed methodology. The validation was performed with sample C-S15-A, as no blank samples were available, and this was the sample with the lowest values of PAs/PANOs found. The validation parameters are presented in Table 2, showing the good analytical performance of the method.

Linearity was evaluated through matrix-matched calibration curves, which were prepared in three consecutive days by spiking the sample extracts with an adequate aliquot of a standard solution containing the target analytes to achieve the desired concentration level of the calibration curve. The calibration was performed at six known concentration levels within the linear range evaluated (Table 2). At the same time, an unspiked sample (denoted blank sample) was also extracted in case some analytes were present in the sample analyzed, so their signal could be subtracted. The curves were constructed by plotting the peak area of each analyte against the analyte concentration and were fitted by linear regression analysis. Good linear regression for all compounds was achieved, obtaining coefficient of determination (R^2^) values ≥0.999. In addition, the linearity coefficient (Cm) was calculated as (1 − (Sd/*m*)) × 100, where Sd is the standard deviation of the calibration slopes obtained on different days and *m* is the average slope, which ranged from 92 to 100 among all the analytes (Table 2), successfully accomplishing the criteria established on the guidelines (≥al 92%) [34,35].

To assess the matrix effect, solvent-based standard calibration curves were also prepared by using working standard solutions (Table S3). The matrix effect was calculated by comparing the slopes of both matrix-matched and solvent-based standard calibration curves (slope matrix-matched/slope solvent-based*100), both expressed in the same units (Table S3). A ratio greater than 100% indicates a signal increase, whereas a ratio lower than 100% means signal suppression due to the adverse effect of the matrix interferences. In the case of a signal suppression or enhancement higher than 20%, matrix effects need to be addressed in calibration [34]. The slope values of the matrix-matched calibration curves were significantly lower than the slopes of the solvent-based calibration curves, leading to ratios lower than 80%, except for echimidine *N*-oxide (111%) (Table 2). Therefore, to quantify the target analytes in the samples, matrix-matched calibration curves should be used to compensate the errors associated with matrix suppression. The method detection limit (MDL) and the method quantification limit (MQL) of each analyte were estimated as the minimum concentration yielding a signal-to-noise ratio (S/N) of 3 and 10, respectively. MDLs ranged from 0.1 to 7.5 µg/kg, and MQLs from 0.5 to 25.0 µg/kg (Table 2).

The accuracy was expressed as the mean recovery obtained from six samples (*n* = 6) spiked with the analytes at a known concentration and subjecting them to the proposed extraction procedure. The accuracy was evaluated at three concentration levels: low (10 µg/kg), medium (100 µg/kg) and high (1000 µg/kg). Recovery values were calculated by comparing the areas of the spiked samples with the areas of simulated samples (samples spiked at the same concentration level but at the end of the extraction procedure prior to their chromatographic analysis). As Table 2 shows, good recovery values for all the analytes were obtained at the three validation levels, as all of them were within the range 70–120% as specified in the recommendations [34,35]. The mean recovery values ranged from 77 to 96% (Table 2).

Method precision (expressed as relative standard deviation percentage, RSD%) was assessed in terms of intraday (repeatability) and interday (reproducibility) precision at the same validation levels as the accuracy. Interday precision was calculated by performing six consecutive injections (*n* = 6) on the same day of a sample spiked at each concentration level tested. Interday precision was evaluated through the analysis of three replicate samples injected in triplicate throughout three different days (*n* = 9) and spiked at each validation level. According to the recommendations, RSD values for precision should be ≤20% for both intra- and interday precision [34,35]. Therefore, satisfactory results were achieved at the three concentration levels evaluated, as the RSD values obtained were lower than 8 and 13% for intra- and interday precision, respectively (Table 2). Overall, the proposed method showed good analytical performance, so it can be successfully used for the extraction and quantification of the PAs and PANOs recommended by the EFSA in oregano samples.

### 3.4. Analysis of Samples

**Figure 4:**
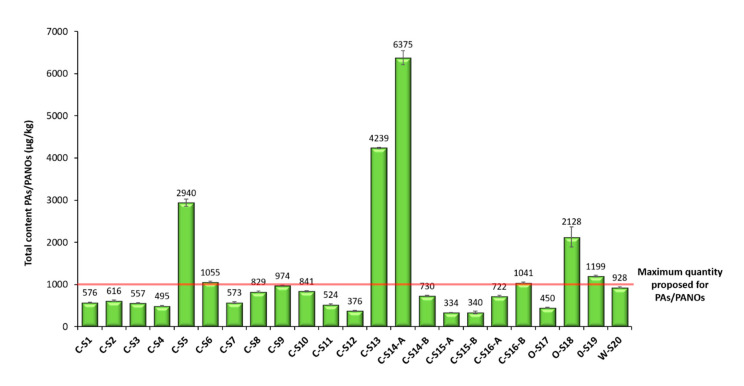
Total content of PAs/PANOs (µg/kg) found in the different oregano samples analyzed by the µ-QuEChERS method proposed. In the sample identification code, the first letter indicates: W for wild farming, O for organic farming and C for conventional farming. Samples ending with an A or B indicate same trademark samples with different lot number.

Finally, the method was applied to the analysis of 23 oregano samples (Figure 4). For quantification, the matrix-matched calibration curves obtained in the validation were used. Contents below the MDL were further treated as 0.0 µg/kg (not detected), and contents between the MDL and the MQL were indicated as <MQL (Table 3). In those cases, in which a high concentration of PAs/PANOs was detected in a sample out of the validated linearity range, the sample extract was properly diluted and reinjected for a new analysis, considering the dilution factor applied. Surprisingly, all the analyzed samples were contaminated with PAs and PANOs, as can be observed in Table 3, although all the 21 target analytes were not always present. Particularly, europine, europine *N*-oxide, lasiocarpine and lasiocarpine *N*-oxide were found to a greater or lesser extent in all the samples analyzed. The average concentration of PAs/PANOs was 1254 µg/kg, which is a notably concerning value despite being lower than the ones reported in previous works (3140 and 6160 µg/kg) [12,36]. Although maximum levels for PAs in food have not yet been stablished, a maximum quantity of 1000 μg/kg for herbs such as oregano is currently under discussion by the EU Commission [36]. In this sense, the great majority of the samples (70%, 16 out of 23) contained between 100 and 1000 µg/kg of PAs/PANOs, whereas the 30% of the samples (7 out of 23) had greater amounts ranging from 1000 to 10,000 µg/kg of PAs/PANOs (Figure 4). The smallest average content was obtained for C-S15-A (340 µg/kg), while the highest amounts were found in C-S14-A (6375 µg/kg) (Figure 4).

PAs can be classified into 4 main families according to their structure and botanical origin:

- Heliotrine-type: including europine, heliotrine, lasiocarpine and their N-oxides.
- Senecionine-type: including erucifoline, jacobine, retrorsine, senecionine, seneciphylline, senecivernine, their N-oxides and senkirkin.
- Lycopsamine-type: including echimidine, indicine, intermedine, lycopsamine and their N-oxides.
- Monocrotaline-type: including monocrotaline, monocrotaline N-oxide and trichodesmine.

In this sense, lasiocarpine, lasiocarpine *N*-oxide, europine, europine *N*-oxide, senecivernine, senecionine, echimidine *N*-oxide, lycopsamine *N*-oxide and intermedine *N*-oxide were the ones which significantly contributed to the contamination of the samples analyzed, as they were often found at relatively higher concentration values (Table 3).

**Figure 5:**
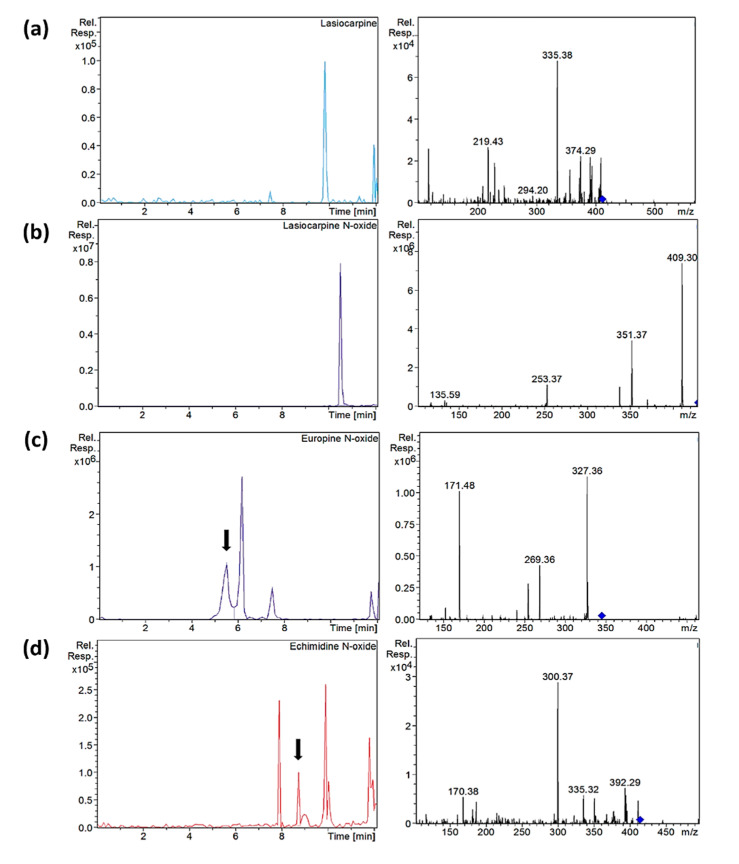
Extracted ion chromatograms and mass spectra (MS^2^) of (**a**) lasiocarpine in sample C-S10, (**b**) lasiocarpine *N*-oxide in sample C-S10, (**c**) europine *N*-oxide in sample O-S9 and (**d**) echimidine *N*-oxide in sample C-S14 after µ-QuEChERS extraction. Chromatographic conditions as Table 1.

Heliotrine-type compounds were found in all the samples analyzed, except heliotrine and its *N*-oxide, which occurred with a lower prevalence. According to the literature, these types of PAs are the most recurrent and frequent in oregano samples, and their presence may be clear evidence of Heliotropium spp. and Borago spp. coharvesting or adulteration [9,12,36]. Among the samples analyzed, C-S13, C-S14-A and O-S18 showed the highest values of heliotrine-type compounds—the *N*-oxides being more predominant than their corresponding PAs (Figure 5)—except C-S13, in which europine, lasiocarpine and heliotrine were more abundant than their *N*-oxides (Table 3).

On the other hand, 9% of the samples (C-S5 and C-S6) mainly contained senecionine-type compounds, mostly senecivernine and senecionine, which were also frequent in the rest of the samples. In this case, senecionine and senecivernine were more predominant than their *N*-oxides (Table 3). The occurrence of these types of PAs is usually associated to the species of the Asteraceae family, such as Senecio vulgaris [12]. Conversely, in 56.5% of the samples (C-S1, C-S7, C-S8, C-S9, C-S11, C-S12, C-S14-B, C-S15-A, C-S15-B, C-S16-A, C-S16-B, O-S19 and W-S20), the most abundant PAs were the lycopsamine-type compounds (Table 3), particularly highlighting the contribution of echimidine *N*-oxide (Figure 5), which presented significant values in almost all the samples analyzed. The content of lycopsamine and intermedine *N*-oxides was also high in the case of C-S1, C-S8, C-S9, C-S16-A, C-S16-B and W-S20, although they were encountered, in general, in a smaller concentration than echimidine *N*-oxide (Table 3). For the lycosamine-type compounds, the *N*-oxides were more predominant than their corresponding PAs, and their occurrence has been associated to the presence of plants belonging to the Boraginaceae family, such as Borago spp. [12]. In contrast, for C-S2, C-S3, C-S4, C-S10 and O-S17, the amount of echimidine *N*-oxide, senecivernine and senecionine was very similar, being the most abundant compounds in these samples (Table 3). These results suggest the wide diversity of unexpected botanical species that could contaminate the oregano plants.

Moreover, samples with different types of farming were evaluated within the samples analyzed. In this sense, samples obtained by wild, organic and conventional farming were included in this work. Oregano belongs to the Lamiaceae family, a plant family for which no formation of PAs has been evidenced in the literature. Therefore, it was expected not to find PAs/PANOs in the wild oregano sample (W-S20), as it was obtained on the branch and its leaves were carefully separated before milling them. Nevertheless, surprisingly, this sample also contained a significative total amount of PAs/PANOs (928 μg/kg) (Figure 4), the lycopsamine-type compounds being the ones which mainly contributed to its contamination, highlighting intermedine *N*-oxide, lycopsamine *N*-oxide and echimidine *N*-oxide (Table 3). Therefore, this may suggest that the contamination of oregano with PAs/PANOs is not only due to the accidental inclusion of PAs-producing foreign plants during harvest or to its intended adulteration, as indicated by other authors [9,12,21,22,36] but also to horizontal natural transfer of PAs/PANOs through the soil [37,38]. Moreover, the total levels of PAs/PANOs found in the wild oregano sample were higher than many of the samples obtained by conventional farming (C-S1, C-S2, C-S3, C-S4, C-S7, C-S8, C-S10, C-S11, C-S12, C-S14-B, C-S15-A, C-S15-B and C-S16-A) (Figure 4). This may suggest that conventional farming intended for commercial oregano production might follow greater controls and good agricultural practices that minimize the presence of weeds in their crops, thus avoiding to some extent the possible contamination routes of PAs/PANOs, such as coharvesting or horizontal natural transfer. Regarding the samples obtained by organic farming (O-S17, O-S18 and O-S19), it was not possible to draw clear conclusions. Among these samples, O-S17 presented the least contamination of PAs and PANOs, with senecionine, senecivernine and echimidine *N*-oxide contributing the most. Conversely, the other organic samples analyzed (O-S18 and 0-S19) showed higher levels exceeding 1000 µg/kg, which were significantly greater than the ones found in the majority of the samples obtained with conventional farming (Figure 4). In O-S18, the contamination was mainly due to europine *N*-oxide, which is an heliotrine-type alkaloid, although significative amounts of senecivernine and senecionine were also found (Table 3). On the other hand, in O-S19, the contamination was mainly due to intermedine and lycopsamine *N*-oxides (Table 3).

Additionally, samples belonging to the same trademark but with a different lot number and acquired in different season periods were also included in this work (C-S14-A, C-S14-B, C-S15-A, C-S15-B, C-S16-A and C-S16-B). In the case of C-S14, significative differences were observed among both samples (Table 3). C-S14-A was the sample with the highest total amount of PAs/PANOs among all the samples analyzed (6375 µg/kg), whereas C-S14-B showed lower levels below 1000 µg/kg (Figure 4). Moreover, the contamination of C-S14-A was mainly due to the occurrence of heliotrine-type alkaloids, while in C-S14-B, the prevalence of lycopsamine-type compounds was higher (Table 3). Conversely, the total levels of C-S15-A and C-S15-B were very similar, and they were the samples with the least occurrence of PAs/PANOs among all the samples analyzed (Figure 4). In addition, for these two samples, the profile of PAs was also very similar, with echimidine *N*-oxide as the main alkaloid found in them (Table 3). On the other hand, C-S16-B showed a slightly higher total amount of PAs than C-S16-A (Figure 4). Nevertheless, the alkaloid profile of both samples was also very similar, with a clear prevalence of lycopsamine-type compounds, stressing the contribution of echimidine, intermedine and lycopsamine *N*-oxides (Table 3). These results suggest that the concentration of PAs for samples belonging to the same trademark may differ with time, but clearly show that contamination with PAs/PANOs is a recurrent issue, independently of time and lot number.

Overall, the results obtained in this work confirm that oregano is highly contaminated with PAs and PANOs, and the sum of the contents found of these compounds should be of concern in view of the potential health risk their intake may cause. Moreover, heliotrine-type alkaloids were always found to a greater or lesser extent in all the samples analyzed. Therefore, this revealed the great importance of monitoring the presence of these contaminants in oregano and the need to further investigate their ocurrence in other matrices in order to establish maximum levels which ensure the safety of consumers.

## 4. Conclusions

A sustainable and green analytical methodology based on the miniaturization of the QuEChERS procedure combined with UHPLC-MS/MS analysis was successfully developed and properly validated to monitor the presence of the 21 PAs and PANOs suggested by the EFSA in different oregano samples. The results obtained showed that the QuEChERS procedure can be effectively scaled down, leading to a significant reduction in solvents and reagents in comparison to the original procedure. Moreover, the feasibility of the method developed was proved through the analysis of different oregano samples. It was revealed that all the samples analyzed were contaminated with PAs and PANOs, and in 30% of the samples, the sum of the total PAs/PANOs exceeded 1000 μg/kg, which is the maximum quantity allowed of these compounds currently under discussion by the EU Commission for aromatic herbs. All the contaminated samples had a coherent pattern of alkaloids. In some of them, the most predominant PAs were the heliotrine-type, while in other samples, the most abundant were the senecionine-type, the lycopsamine-type or both types. In this sense, lasiocarpine, lasiocarpine *N*-oxide, europine, europine *N*-oxide, senecivernine, senecionine, echimidine *N*-oxide, lycopsamine *N*-oxide and intermedine *N*-oxide were the ones which significantly contributed to the contamination of the samples analyzed, as they were often found at relatively higher concentration values. This wide diversity of PAs suggests that oregano plants may be contaminated with a great variety of foreign plants growing among the oregano cultivation belonging to the Boraginaceae and Asteraceae families, leading to their coharvest. Nevertheless, the analysis of a wild oregano sample on the branch also showed contamination, indicating another possible contamination route of PAs by horizontal natural transfer through the soil. Nowadays, the EFSA only recommends a set of 17 PAs/PANOs to be monitored in food items, excluding europine, heliotrine and their corresponding *N*-oxides, although the inclusion of these compounds is currently under consideration. In light of our results, the monitorization of these heliotrine-type compounds is of outmost importance, as their occurrence was detected in almost all the samples analyzed, particularly europine and europine *N*-oxide. Overall, it can be confirmed that oregano is highly contaminated with concerning values of PAs and PANOs that may entail potential health risks for consumers. Therefore, the results obtained in this work reveal the great need to monitor and regulate the presence of these compounds in aromatic herbs in order to establish maximum concentration levels and, thus, ensure food safety.

## Figures and Tables

**Table 1:** Retention time and mass spectrum parameters of the targeted analytes using the UHPLC-IT-MS/MS method developed.

Analyte	Retention Time (min)	Ionization Mode	Precursor Ion (*m/z*)	Fragmentation Amplitude	MS^2^. Product Ions ^a^ (*m/z*)
Intermedine	5.6	ESI (+)	299	0.70	138 *, 120
Europine	5.7	ESI (+)	329	0.80	253 *, 138
Lycopsamine	5.8	ESI (+)	299	0.70	138 *, 120
Europine *N*-oxide	6.2	ESI (+)	345	0.80	327 *, 171.5
Intermedine *N*-oxide	6.4	ESI (+)	315	0.80	225, 171.5 *
Lycopsamine *N*-oxide	6.5	ESI (+)	315	0.80	171.5 *, 138
Retrorsine	6.8	ESI (+)	351	0.80	323 *, 275
Retrorsine *N*-oxide	7.0	ESI (+)	367	0.90	339 *, 245
Seneciphylline	7.2	ESI (+)	333	0.80	305 *, 120
Heliotrine	7.2	ESI (+)	313.5	0.70	138 *, 120
Seneciphylline *N*-oxide	7.5	ESI (+)	350	0.80	321 *, 118
Heliotrine *N*-oxide	7.6	ESI (+)	329	1.00	171 *, 136
Senecivernine	7.9	ESI (+)	335	0.80	307 *, 120
Senecionine	7.9	ESI (+)	335	0.80	307 *, 120
Senecivernine *N*-oxide	8.1	ESI (+)	351	0.80	323 *, 219.5
Senecionine *N*-oxide	8.3	ESI (+)	352	1.00	220, 118 *
Echimidine	8.7	ESI (+)	398	0.60	220, 120 *
Echimidine *N*-oxide	8.7	ESI (+)	413	0.70	395 *, 351
Senkirkin	9.1	ESI (+)	365	0.80	167.5 *, 150
Lasiocarpine	9.8	ESI (+)	411	0.70	335 *, 219.5
Lasiocarpine *N*-oxide	10.4	ESI (+)	428	0.80	409 *, 352

^a^ Predominant product ion. * Ions used for quantification. Isolation width (*m*/*z*) is 4. Chromatographic conditions with the optimized gradient elution: 5% B (0–0.5 min), 5–50% B (0.5–7 min), 50% B (7–7.5 min), 50–100% B (7.5–11 min), 100% B (11–12 min), 100–5% B (12–14 min). Water containing 0.2% formic acid and 5 mM ammonium acetate as mobile phase A and methanol containing 10 mM ammonium acetate as mobile phase B. The flow rate was 0.25 mL/min.

**Table 2:** Validation parameters of the µ-QuEChERS method for the determination of the target PAs/PANOs in oregano samples.

Analytes	Linear Range (µg/kg)	Matrix-Matched Calibration R^2^/cm	Accuracy		Precision		MDL (µg/kg)	MQL (µg/kg)	ME (%)
			Recovery (% ± sd)	Mean Recovery (% ± sd)	Intra-Day Precision (RSD%)	Inter-Day Precision (RSD%)			
Intermedine	25.0–500.0	*y* = 2035*x* + 5771 0.999/93	78 ± 7 ^a^	77 ± 3	5 ^a^	6 ^a^	7.5	25.0	23
			74 ± 2 ^b^		7 ^b^	10 ^b^			
			80 ± 6 ^c^		3 ^c^	3 ^c^			
Europine	10.0–500.0	*y* = 4482*x* + 58,782 0.999/98	83 ± 4 ^a^	83 ± 7	4 ^a^	7 ^a^	3.0	10.0	23
			77 ± 2 ^b^		5 ^b^	6 ^b^			
			90 ± 11 ^c^		4 ^c^	5 ^c^			
Lycopsamine	22.0–500.0	*y* = 1559*x* + 8916 0.999/99	95 ± 7 ^a^	90 ± 13	7 ^a^	12 ^a^	6.7	22.0	18
			75 ± 7 ^b^		5 ^b^	8 ^b^			
			99 ± 7 ^c^		1 ^c^	4 ^c^			
Europine *N*-oxide	7.5–500.0	*y* = 6816*x* + 43,628 0.999/100	100 ± 5 ^a^	88 ± 13	6 ^a^	8 ^a^	2.2	7.5	16
			75 ± 6 ^b^		5 ^b^	9 ^b^			
			90 ± 5 ^c^		3 ^c^	5 ^c^			
Intermedine *N*-oxide	7.5–500.0	*y* = 2111*x* + 75,308 0.999/93	78 ± 3 ^a^	80 ± 11	8 ^a^	12 ^a^	2.2	7.5	17
			71 ± 2 ^b^		7 ^b^	11 ^b^			
			92 ± 4 ^c^		2 ^c^	4 ^c^			
Lycopsamine *N*-oxide	12.5–500.0	*y* = 2017*x* + 29,990 0.999/93	94 ± 3 ^a^	86 ± 13	5 ^a^	8 ^a^	3.7	12.5	13
			71 ± 1 ^b^		4 ^b^	5 ^b^			
			92 ± 3 ^c^		7 ^c^	10 ^c^			
Retrorsine	5.5–500.0	*y* = 1091*x* + 12,358 0.999/97	91 ± 4 ^a^	82 ± 9	7 ^a^	11 ^a^	1.7	5.5	13
			73 ± 5 ^b^		4 ^b^	12 ^b^			
			82 ± 5 ^c^		2 ^c^	4 ^c^			
Retrorsine *N*-oxide	3.5–500.0	*y* = 543*x* + 13,424 0.999/95	79 ± 1 ^a^	83 ± 5	6 ^a^	8 ^a^	1.0	3.5	18
			81 ± 8 ^b^		3 ^b^	7 ^b^			
			89 ± 6 ^c^		5	7			
Seneciphylline	2.0–500.0	*y* = 2244*x* + 25,457 0.999/94	88 ± 7 ^a^	90 ± 4	7 ^a^	9 ^a^	0.7	2.0	12
			95 ± 5 ^b^		3 ^b^	10 ^b^			
			87 ± 7 ^c^		4 ^c^	5 ^c^			
Heliotrine	4.0–500.0	*y* = 4771*x* + 9413 0.999/92	80 ± 9 ^a^	91 ± 9	3 ^a^	8 ^a^	1.3	4.0	17
			96 ± 4 ^b^		7 ^b^	9 ^b^			
			96 ± 12 ^c^		2 ^c^	6 ^c^			
Seneciphylline *N*-oxide	1.0–250.0	*y* = 2856*x* – 29,835 0.999/95	95 ± 4 ^a^	88 ± 10	3 ^a^	4 ^a^	0.4	1.0	34
			77 ± 5 ^b^		6 ^b^	9 ^b^			
			93 ± 7 ^c^		2 ^c^	7 ^c^			
Heliotrine *N*-oxide	3.0–500.0	*y* = 684*x* + 13,923 0.999/95	81 ± 7 ^a^	87 ± 1	8 ^a^	9 ^a^	1.0	3.0	3
			82 ± 5 ^b^		8 ^b^	12 ^b^			
			99 ± 10 ^c^		5 ^c^	12 ^c^			
Senecivernine	0.5–500.0	*y* = 7802*x* + 480,780 0.999/96	98 ± 7 ^a^	89 ± 13	4 ^a^	6 ^a^	0.1	0.5	25
			74 ± 3 ^b^		4 ^b^	9 ^b^			
			95 ± 8 ^c^		2 ^c^	4 ^c^			
Senecionine	0.5–500.0	*y* = 7830*x* + 339,046 0.999/95	99 ± 4 ^a^	88 ± 12	4 ^a^	7 ^a^	0.1	0.5	18
			76 ± 6 ^b^		4 ^b^	7 ^b^			
			90 ± 11 ^c^		4 ^c^	6 ^c^			
Senecivernine *N*-oxide	6.0–250.0	*y* = 2287*x* + 4421 0.999/97	95 ± 2 ^a^	96 ± 4	6 ^a^	10 ^a^	1.8	6.0	39
			100 ± 6 ^b^		6 ^b^	7 ^b^			
			93 ± 6 ^c^		4 ^c^	8 ^c^			
Senecionine *N*-oxide	3.0–500.0	*y* = 1208*x* + 146,398 0.999/98	96 ± 8 ^a^	92 ± 4	8 ^a^	13 ^a^	0.9	3.0	28
			93 ± 8 ^b^		3 ^b^	5 ^b^			
			88 ± 5 ^c^		6 ^c^	8 ^c^			
Echimidine	7.0–500.0	*y* = 4704*x* + 167,863 0.999/94	78 ± 4 ^a^	82 ± 10	5 ^a^	5 ^a^	2.0	7.0	17
			75 ± 2 ^b^		6 ^b^	12 ^b^			
			94 ± 11 ^c^		4 ^c^	6 ^c^			
Echimidine *N*-oxide	7.5–250.0	*y* = 1005*x* – 48,988 0.999/96	87 ± 2 ^a^	86 ± 2	8 ^a^	12 ^a^	2.0	7.5	111
			86 ± 10 ^b^		7 ^b^	9 ^b^			
			84 ± 11 ^c^		4 ^c^	12 ^c^			
Senkirkin	7.5–500.0	*y* = 1327*x* + 17,984 0.999/97	74 ± 5 ^a^	83 ± 15	3 ^a^	7 ^a^	2.0	7.5	8
			75 ± 3 ^b^		4 ^b^	7 ^b^			
			101 ± 2 ^c^		5 ^c^	7 ^c^			
Lasiocarpine	25.0–500.0	*y* = 282*x* + 4928 0.999/92	96 ± 5 ^a^	94 ± 2	7 ^a^	10 ^a^	7.5	25.0	18
			95 ± 3 ^b^		3 ^b^	10 ^b^			
			92 ± 10 ^c^		5 ^c^	7 ^c^			
Lasiocarpine *N*-oxide	10.0–500.0	*y* = 6252*x* + 2254 0.999/100	101 ± 6 ^a^	92 ± 10	5 ^a^	12 ^a^	3.0	10.0	13
			93 ± 7 ^b^		8 ^b^	10 ^b^			
			82 ± 7 ^c^		5 ^c^	5 ^c^			

Cm: linearity coefficient calculated as (1 − (Sd/*m*)) × 100, where Sd is the standard deviation of the calibration slopes obtained on different days and *m* is the average slope; Recovery: mean recovery obtained from six samples (*n* = 6) spiked with the analytes at a known concentration level, and subjected to the proposed extraction procedure; Intraday precision: six consecutive injections (*n* = 6) on the same day of a sample spiked with the analytes at a known concentration level; Interday precision: three replicates of a sample injected in triplicate throughout three different days (*n* = 9) and spiked with the analytes at a known concentration level; MDL: method detection limit; MQL: method quantification limit; ME: matrix effect. ^a^ Low spiked level (10 µg/kg); ^b^ Medium spiked level (100 µg/kg); ^c^ High spiked level (1000 µg/kg).

**Table 3:** Content of the target PAs/PANOs (µg/kg) quantified in the different oregano samples analyzed by the µ-QuEChERS method proposed.

Analytes (µg/kg)	C-S1	C-S2	C-S3	C-S4	C-S5	C-S6	C-S7	C-S8	C-S9	C-S10	C-S11	
Intermedine	n.d.	n.d.	n.d.	<MQL	<MQL	n.d.	n.d.	n.d.	n.d.	n.d.	n.d.	
Europine	<MQL	<MQL	<MQL	<MQL	<MQL	<MQL	<MQL	<MQL	<MQL	<MQL	<MQL	
Lycopsamine	n.d.	n.d.	n.d.	n.d.	<MQL	<MQL	n.d.	<MQL	n.d.	n.d.	n.d.	
Europine *N*-oxide	14 ± 3$\;\begin{array}{c} c\\ a \end{array}$	<MQL	<MQL	<MQL	25 ± 5$\;\begin{array}{c} a,b\\ a \end{array}$	15 ± 4$\;\begin{array}{c} a,b\\ a \end{array}$	14 ± 3$\;\begin{array}{c} c\\ a \end{array}$	<MQL	16 ± 6$\;\begin{array}{c} c,d\\ a \end{array}$	15 ± 4$\;\begin{array}{c} d,e\\ a \end{array}$	8 ± 2$\;\begin{array}{c} b\\ a \end{array}$	
Intermedine *N*-oxide	93 ± 4$\;\begin{array}{c} g\\ b \end{array}$	n.d.	n.d.	n.d.	n.d.	n.d.	n.d.	138 ± 4$\;\begin{array}{c} f\\ c \end{array}$	235 ± 5$\;\begin{array}{c} g\\ d \end{array}$	n.d.	n.d.	
Lycopsamine *N*-oxide	111 ± 5$\;\begin{array}{c} h\\ e \end{array}$	n.d.	<MQL	n.d.	39 ± 2$\;\begin{array}{c} b,c\\ b \end{array}$	<MQL	<MQL	165 ± 7$\;\begin{array}{c} g\\ f \end{array}$	261 ± 11$\;\begin{array}{c} h\\ h \end{array}$	<MQL	17 ± 2$\;\begin{array}{c} c\\ a \end{array}$	
Retrorsine	10 ± 2$\begin{array}{c} b,c\\ a \end{array}$	<MQL	n.d.	n.d.	17 ± 1$\;\begin{array}{c} a,b\\ e,f \end{array}$	10 ± 5$\;\begin{array}{c} a\\ a,b,c \end{array}$	6.7 ± 0.4$\;\begin{array}{c} a,b\\ a \end{array}$	<MQL	12 ± 4$\;\begin{array}{c} b,c\\ c,d \end{array}$	<MQL	<MQL	
Retrorsine *N*-oxide	5 ± 2$\;\begin{array}{c} a\\ a,b \end{array}$	6 ± 2$\;\begin{array}{c} b\\ a,b,c \end{array}$	<MQL	n.d.	41 ± 12$\;\begin{array}{c} b,c\\ g \end{array}$	12 ± 4$\;\begin{array}{c} a\\ b,c,d,e \end{array}$	55 ± 5$\;\begin{array}{c} f\\ h \end{array}$	19 ± 3$\;\begin{array}{c} b,c\\ e \end{array}$	10 ± 3$\;\begin{array}{c} b,c\\ b,c,d \end{array}$	<MQL	31 ± 4$\;\begin{array}{c} d\\ f \end{array}$	
Seneciphylline	n.d.	n.d.	n.d.	n.d.	12 ± 2$\;\begin{array}{c} a,b\\ b \end{array}$	n.d.	<MQL	n.d.	n.d.	5 ± 4$\;\begin{array}{c} a,b\\ a \end{array}$	n.d.	
Heliotrine	5 ± 2$\;\begin{array}{c} a\\ a,b \end{array}$	5 ± 1$\;\begin{array}{c} a,b\\ a,b \end{array}$	7.9 ± 0.3$\;\begin{array}{c} a\\ b,c \end{array}$	<MQL	22 ± 5$\;\begin{array}{c} a,b\\ e \end{array}$	8 ± 2$\;\begin{array}{c} a\\ b,c \end{array}$	36 ± 5$\;\begin{array}{c} e\\ g \end{array}$	<MQL	<MQL	9.3 ± 0.4$\;\begin{array}{c} b,c\\ b,c \end{array}$	6.1 ± 0.5$\;\begin{array}{c} a,b\\ b \end{array}$	
Seneciphylline *N*-oxide	18.7 ± 0.8$\;\begin{array}{c} d\\ a,b \end{array}$	82 ± 3$\;\begin{array}{c} f\\ e \end{array}$	20.5 ± 0.7$\;\begin{array}{c} b\\ a,b \end{array}$	26 ± 1$\;\begin{array}{c} c\\ b \end{array}$	88 ± 18$\;\begin{array}{c} d\\ e \end{array}$	19 ± 4$\;\begin{array}{c} b\\ a,b \end{array}$	65 ± 7$\;\begin{array}{c} g\\ d \end{array}$	100 ± 13$\;\begin{array}{c} e\\ f \end{array}$	19 ± 2$\;\begin{array}{c} d,e\\ a,b \end{array}$	53 ± 4$\;\begin{array}{c} f\\ c \end{array}$	49 ± 6$\;\begin{array}{c} e\\ c \end{array}$	
Heliotrine *N*-oxide	13.2 ± 0.9$\begin{array}{c} c\\ b,c \end{array}$	n.d.	n.d.	n.d.	47 ± 1$\;\begin{array}{c} b,c\\ e \end{array}$	n.d.	15 ± 6$\;\begin{array}{c} c\\ c \end{array}$	6.6 ± 0.4$\;\begin{array}{c} a\\ a \end{array}$	n.d.	n.d.	n.d.	
Senecivernine	11 ± 4$\;\begin{array}{c} b,c\\ a \end{array}$	154 ± 3$\;\begin{array}{c} h\\ e \end{array}$	102 ± 7$\;\begin{array}{c} d\\ d \end{array}$	105 ± 4$\;\begin{array}{c} e\\ d \end{array}$	1027 ± 58$\;\begin{array}{c} f\\ i \end{array}$	385 ± 6$\;\begin{array}{c} e\\ g \end{array}$	66 ± 3$\;\begin{array}{c} g\\ b,c \end{array}$	6 ±$\;\begin{array}{c} a\\ a \end{array}$ 5	6 ± 3$\;\begin{array}{c} a,b\\ a \end{array}$	225.4 ± 0.4$\;\begin{array}{c} i\\ f \end{array}$	n.d.	
Senecionine	24 ± 3$\;\begin{array}{c} e\\ a,b \end{array}$	166 ± 4$\;\begin{array}{c} i\\ e \end{array}$	120 ± 5$\;\begin{array}{c} e\\ d \end{array}$	121 ± 8$\;\begin{array}{c} f\\ d \end{array}$	1103 ± 60	385 ± 15$\;\begin{array}{c} e\\ g \end{array}$	81.8 ± 0.8$\;\begin{array}{c} h\\ c \end{array}$	24 ± 5$\;\begin{array}{c} c\\ a,b \end{array}$	24 ± 3$\;\begin{array}{c} e\\ a,b \end{array}$	231 ± 1$\;\begin{array}{c} j\\ f \end{array}$	2.4 ± 0.3$\;\begin{array}{c} a,b\\ a \end{array}$	
Senecivernine *N*-oxide	8 ± 1$\;\begin{array}{c} a,b\\ a,b \end{array}$	17 ± 3$\;\begin{array}{c} c\\ d,e \end{array}$	10.9 ± 0.3$\;\begin{array}{c} a\\ b,c \end{array}$	8 ± 2$\;\begin{array}{c} a\\ a,b \end{array}$	138 ± 4$\;\begin{array}{c} e\\ j \end{array}$	11 ± 1$\;\begin{array}{c} a\\ b,c \end{array}$	24 ± 6$\;\begin{array}{c} d\\ f \end{array}$	53 ± 3$\;\begin{array}{c} d\\ h \end{array}$	9 ± 3$\;\begin{array}{c} b,c\\ a,b,c \end{array}$	10.8 ± 0.7$\;\begin{array}{c} c,d\\ a,b,c \end{array}$	70 ± 4$\;\begin{array}{c} g\\ i \end{array}$	
Senecionine *N*-oxide	n.d.	16 ± 3$\;\begin{array}{c} c\\ b \end{array}$	n.d.	n.d.	91 ± 9$\;\begin{array}{c} d\\ c \end{array}$	n.d.	9 ± 5$\;\begin{array}{c} b,c\\ a \end{array}$	n.d.	n.d.	n.d.	n.d.	
Echimidine	<MQL	<MQL	<MQL	<MQL	19 ± 5$\;\begin{array}{c} a,b\\ a \end{array}$	<MQL	<MQL	<MQL	n.d.	<MQL	n.d.	
Echimidine *N*-oxide	160 ± 2$\;\begin{array}{c} i\\ e,f \end{array}$	100 ± 6$\;\begin{array}{c} g\\ a,b,c \end{array}$	194 ± 2$\;\begin{array}{c} f\\ h,i \end{array}$	185 ± 2$\;\begin{array}{c} g\\ g,h \end{array}$	140 ± 4$\;\begin{array}{c} e\\ d \end{array}$	148 ± 1$\;\begin{array}{c} d\\ d,e \end{array}$	106 ± 7$\;\begin{array}{c} i\\ c \end{array}$	253 ± 6$\;\begin{array}{c} h\\ k \end{array}$	302 ± 7$\;\begin{array}{c} i\\ l \end{array}$	202 ± 11$\;\begin{array}{c} h\\ i,j \end{array}$	267 ± 10$\;\begin{array}{c} h\\ k \end{array}$	
Senkirkin	10.32 ± 0.06$\;\begin{array}{c} b,c\\ a,b \end{array}$	n.d.	8 ± 2$\;\begin{array}{c} a\\ a \end{array}$	n.d.	27 ± 5$\;\begin{array}{c} a,b\\ e \end{array}$	<MQL	10 ± 1$\;\begin{array}{c} b,c\\ a,b \end{array}$	<MQL	<MQL	<MQL	<MQL	
Lasiocarpine	81 ± 3$\;\begin{array}{c} f\\ d \end{array}$	45 ± 5$\;\begin{array}{c} e\\ a,b,c,d \end{array}$	76 ± 3$\;\begin{array}{c} c\\ b,c,d \end{array}$	36 ± 2$\;\begin{array}{c} d\\ a,b \end{array}$	66 ± 4$\;\begin{array}{c} c,d\\ a,b,c,d \end{array}$	47 ± 4$\;\begin{array}{c} c\\ a,b,c,d \end{array}$	57 ± 7$\;\begin{array}{c} f\\ a,b,c,d \end{array}$	50 ± 5$\;\begin{array}{c} d\\ a,b,c,d \end{array}$	65 ± 4$\;\begin{array}{c} f\\ a,b,c,d \end{array}$	72 ± 7$\;\begin{array}{c} g\\ a,b,c,d \end{array}$	55 ± 7$\;\begin{array}{c} f\\ a,b,c,d \end{array}$	
Lasiocarpine *N*-oxide	12 ± 1$\;\begin{array}{c} b,c\\ a \end{array}$	25 ± 5$\;\begin{array}{c} d\\ a,b \end{array}$	18 ± 2$\;\begin{array}{c} b\\ a,b \end{array}$	14 ± 1$\;\begin{array}{c} b\\ a,b \end{array}$	38 ± 3$\;\begin{array}{c} b,c\\ a,b,c \end{array}$	15 ± 4$\;\begin{array}{c} a,b\\ a,b \end{array}$	27 ± 6$\;\begin{array}{c} d\\ a,b \end{array}$	14 ± 2$\;\begin{array}{c} b\\ a,b \end{array}$	15 ± 4$\;\begin{array}{c} c,d\\ a,b \end{array}$	17.9 ± 0.2$\;\begin{array}{c} e\\ a,b \end{array}$	18 ± 1$\;\begin{array}{c} c\\ a,b \end{array}$	
Total	576 ± 10	616 ± 12	557 ± 10	495 ± 10	2940 ± 88	1055 ± 19	573 ± 19	829 ± 19	974 ± 18	841 ± 15	524 ± 15	
**Analytes (µg/kg)**	**C-S12**	**C-S13**	**C-S14-A**	**C-S14-B**	**C-S15-A**	**C-S15-B**	**C-S16-A**	**C-S16-B**	**O-S17**	**O-S18**	**0-S19**	**W-S20**
Intermedine	<MQL	47 ± 3$\;\begin{array}{c} f\\ b \end{array}$	27.7 ± 0.8$\;\begin{array}{c} a,b\\ a \end{array}$	<MQL	<MQL	n.d.	<MQL	n.d.	n.d.	<MQL	n.d.	n.d.
Europine	<MQL	3142 ± 4$\;\begin{array}{c} j\\ d \end{array}$	170 ± 3$\;\begin{array}{c} c\\ c \end{array}$	<MQL	<MQL	11 ± 2$\;\begin{array}{c} a\\ a \end{array}$	<MQL	<MQL	<MQL	25 ± 6$\;\begin{array}{c} a,b\\ b \end{array}$	<MQL	<MQL
Lycopsamine	<MQL	44 ± 5$\;\begin{array}{c} f\\ b \end{array}$	25.4 ± 0.8$\;\begin{array}{c} a,b\\ a \end{array}$	<MQL	<MQL	n.d.	<MQL	n.d.	n.d.	<MQL	n.d.	<MQL
Europine *N*-oxide	9 ± 1$\;\begin{array}{c} c,d\\ a \end{array}$	118 ± 7$\;\begin{array}{c} h\\ b \end{array}$	737 ± 9$\;\begin{array}{c} e\\ c \end{array}$	12.2 ± 0.2$\;\begin{array}{c} a,b\\ a \end{array}$	10.5 ± 0.1$\;\begin{array}{c} c\\ a \end{array}$	23 ± 4$\;\begin{array}{c} b\\ a \end{array}$	16.6 ± 0.8$\;\begin{array}{c} c,d\\ a \end{array}$	11.3 ± 0.2$\;\begin{array}{c} a\\ a \end{array}$	17 ± 1$\;\begin{array}{c} c\\ a \end{array}$	1195 ± 235$\;\begin{array}{c} d\\ d \end{array}$	10.1 ± 0.2$\;\begin{array}{c} b,c\\ a \end{array}$	10.5 ± 0.7$\;\begin{array}{c} b\\ a \end{array}$
Intermedine *N*-oxide	n.d.	n.d.	22.8 ± 0.1$\;\begin{array}{c} a,b\\ a \end{array}$	n.d.	n.d.	n.d.	152 ± 7$\;\begin{array}{c} i\\ c \end{array}$	206 ± 1$\;\begin{array}{c} d\\ d \end{array}$	n.d.	n.d.	602 ± 8$\;\begin{array}{c} h\\ f \end{array}$	305 ± 10$\;\begin{array}{c} i\\ e \end{array}$
Lycopsamine *N*-oxide	24 ± 4$\;\begin{array}{c} g\\ a \end{array}$	18 ± 4$\;\begin{array}{c} b,c,d\\ a \end{array}$	78 ± 4$\;\begin{array}{c} b\\ c \end{array}$	104 ± 9$\;\begin{array}{c} h\\ d,e \end{array}$	<MQL	40 ± 3$\;\begin{array}{c} c\\ b \end{array}$	99 ± 5$\;\begin{array}{c} h\\ d \end{array}$	99 ± 6$\;\begin{array}{c} c\\ d \end{array}$	n.d.	35.1 ± 0.3$\;\begin{array}{c} a,b\\ b \end{array}$	211 ± 10$\;\begin{array}{c} g\\ g \end{array}$	163 ± 7$\;\begin{array}{c} g\\ f \end{array}$
Retrorsine	19 ± 2$\;\begin{array}{c} f\\ f \end{array}$	12 ± 2$\;\begin{array}{c} a,b\\ c,d \end{array}$	19 ± 2$\;\begin{array}{c} a,b\\ f \end{array}$	9 ± 3$\;\begin{array}{c} a\\ a,b,c \end{array}$	14 ± 2$\;\begin{array}{c} a\\ d,e \end{array}$	8 ± 2$\;\begin{array}{c} c\\ a,b \end{array}$	11 ± 2$\;\begin{array}{c} b,c\\ b,c,d \end{array}$	10 ± 3$\;\begin{array}{c} a\\ a,b,c \end{array}$	<MQL	<MQL	<MQL	<MQL
Retrorsine *N*-oxide	12.8 ± 0.8$\;\begin{array}{c} d,e\\ c,d,e \end{array}$	46 ± 7$\;\begin{array}{c} f\\ g \end{array}$	33 ± 9$\;\begin{array}{c} a,b\\ f \end{array}$	15 ± 3$\;\begin{array}{c} b,c\\ d,e \end{array}$	5.4 ± 0.4$\;\begin{array}{c} a\\ a,b,c \end{array}$	4.8 ± 0.8$\;\begin{array}{c} c\\ a,b \end{array}$	5 ± 1$\;\begin{array}{c} a,b\\ a,b \end{array}$	n.d.	n.d.	<MQL	n.d.	7 ± 2$\;\begin{array}{c} b\\ a,b,c \end{array}$
Seneciphylline	n.d.	n.d.	n.d.	n.d.	n.d.	n.d.	n.d.	n.d.	n.d.	n.d.	n.d.	n.d.
Heliotrine	12 ± 2$\;\begin{array}{c} c,d,e\\ c \end{array}$	30 ± 4$\;\begin{array}{c} e\\ f \end{array}$	45 ± 10$\;\begin{array}{c} a,b\\ h \end{array}$	<MQL	6.2 ± 0.9$\;\begin{array}{c} a,b\\ b \end{array}$	n.d.	12 ± 1$\;\begin{array}{c} c\\ c \end{array}$	5 ± 1$\;\begin{array}{c} a\\ a,b \end{array}$	6.6 ± 0.5$\;\begin{array}{c} a\\ b \end{array}$	8.1 ± 0.7$\;\begin{array}{c} a,b\\ b,c \end{array}$	7 ± 1$\;\begin{array}{c} a,b\\ b,c \end{array}$	17 ± 1$\;\begin{array}{c} c\\ d \end{array}$
Seneciphylline *N*-oxide	17 ± 1$\;\begin{array}{c} e,f\\ a,b \end{array}$	20 ± 3$\;\begin{array}{c} c,d\\ a,b \end{array}$	23 ± 4$\;\begin{array}{c} a,b\\ a,b \end{array}$	22 ± 3$\;\begin{array}{c} d\\ a,b \end{array}$	23 ± 4$\;\begin{array}{c} e\\ a,b \end{array}$	n.d.	21 ± 2$\;\begin{array}{c} d\\ a,b \end{array}$	20 ± 4$\;\begin{array}{c} a\\ a,b \end{array}$	13.7 ± 0.2$\;\begin{array}{c} b,c\\ a \end{array}$	19 ± 2$\;\begin{array}{c} a,b\\ a,b \end{array}$	16 ± 3$\;\begin{array}{c} c\\ a,b \end{array}$	20 ± 3$\;\begin{array}{c} c\\ a,b \end{array}$
Heliotrine *N*-oxide	n.d.	<MQL	34 ± 4$\;\begin{array}{c} a,b\\ d \end{array}$	n.d.	n.d.	n.d.	<MQL	6 ± 2$\;\begin{array}{c} a\\ a \end{array}$	n.d.	75 ± 6$\;\begin{array}{c} a,b\\ f \end{array}$	11 ± 4$\;\begin{array}{c} b,c\\ b \end{array}$	n.d.
Senecivernine	n.d.	n.d.	492 ± 78$\;\begin{array}{c} d\\ h \end{array}$	94 ± 5$\;\begin{array}{c} g\\ c,d \end{array}$	n.d.	8 ± 0.7$\;\begin{array}{c} a\\ a \end{array}$	19 ± 2$\;\begin{array}{c} d\\ a \end{array}$	21 ± 4$\;\begin{array}{c} a\\ a \end{array}$	115 ± 7$\;\begin{array}{c} g\\ d \end{array}$	224 ± 7$\;\begin{array}{c} c\\ f \end{array}$	57 ± 4$\;\begin{array}{c} e\\ b \end{array}$	n.d.
Senecionine	4 ± 1$\;\begin{array}{c} a,b\\ a \end{array}$	24 ± 4$\;\begin{array}{c} d,e\\ a,b \end{array}$	524 ± 80$\;\begin{array}{c} d\\ h \end{array}$	52 ± 4$\;\begin{array}{c} f\\ b,c \end{array}$	5 ± 1$\;\begin{array}{c} a\\ a \end{array}$	n.d.	60 ± 6$\;\begin{array}{c} f\\ b,c \end{array}$	62 ± 4$\;\begin{array}{c} b\\ b,c \end{array}$	126 ± 8$\;\begin{array}{c} h\\ d \end{array}$	240 ± 6$\;\begin{array}{c} c\\ f \end{array}$	82 ± 5$\;\begin{array}{c} f\\ c \end{array}$	5.7 ± 0.4$\;\begin{array}{c} a,b\\ a \end{array}$
Senecivernine *N*-oxide	7.2 ± 0.6$\;\begin{array}{c} b,c\\ a,b \end{array}$	17 ± 2$\;\begin{array}{c} b,c\\ d,e \end{array}$	11 ± 2$\;\begin{array}{c} a,b\\ b,c \end{array}$	13 ± 2$\;\begin{array}{c} a,b\\ c,d \end{array}$	10.8 ± 0.2$\;\begin{array}{c} c\\ a,b,c \end{array}$	n.d.	12 ± 2$\;\begin{array}{c} c\\ b,c \end{array}$	20 ± 5$\;\begin{array}{c} a\\ e,f \end{array}$	<MQL	6 ± 2$\;\begin{array}{c} a,b\\ a \end{array}$	13 ± 2$\;\begin{array}{c} b,c\\ c,d \end{array}$	29 ± 1$\;\begin{array}{c} d\\ g \end{array}$
Senecionine *N*-oxide	n.d.	n.d.	n.d.	n.d.	n.d.	n.d.	n.d.	n.d.	n.d.	n.d.	n.d.	n.d.
Echimidine	n.d.	n.d.	n.d.	n.d.	n.d.	<MQL	n.d.	n.d.	n.d.	n.d.	n.d.	n.d.
Echimidine *N*-oxide	186 ± 3$\;\begin{array}{c} i\\ g,h \end{array}$	322 ± 7$\;\begin{array}{c} i\\ m \end{array}$	183 ± 11$\;\begin{array}{c} c\\ g,h \end{array}$	297 ± 5$\;\begin{array}{c} i\\ l \end{array}$	204 ± 4$\;\begin{array}{c} g\\ i,j \end{array}$	173 ± 29$\;\begin{array}{c} d\\ f,g \end{array}$	182 ± 9$\;\begin{array}{c} j\\ g,h \end{array}$	491 ± 11$\;\begin{array}{c} e\\ n \end{array}$	102 ± 7$\;\begin{array}{c} f\\ b,c \end{array}$	84.6 ± 0.9$\;\begin{array}{c} a,b\\ a \end{array}$	87 ± 6$\;\begin{array}{c} f\\ a,b \end{array}$	211 ± 8$\;\begin{array}{c} h\\ j \end{array}$
Senkirkin	<MQL	9.8 ± 0.8$\;\begin{array}{c} a\\ a \end{array}$	17 ± 2$\;\begin{array}{c} a,b\\ c,d \end{array}$	19 ± 3$\;\begin{array}{c} c,d\\ d \end{array}$	8.6 ± 0.1$\;\begin{array}{c} b,c\\ a \end{array}$	9 ± 2$\;\begin{array}{c} a\\ a \end{array}$	16 ± 2$\;\begin{array}{c} c,d\\ c \end{array}$	10 ± 3$\;\begin{array}{c} a\\ a,b \end{array}$	10.5 ± 0.1$\;\begin{array}{c} a,b\\ a,b \end{array}$	105 ± 2$\;\begin{array}{c} b\\ g \end{array}$	13 ± 1$\;\begin{array}{c} b,c\\ b \end{array}$	30 ± 1$\;\begin{array}{c} d\\ f \end{array}$
Lasiocarpine	70 ± 11$\;\begin{array}{c} h\\ a,b,c,d \end{array}$	326 ± 1$\;\begin{array}{c} i\\ e \end{array}$	986 ± 98$\;\begin{array}{c} f\\ f \end{array}$	48 ± 4$\;\begin{array}{c} e,f\\ a,b,c,d \end{array}$	36 ± 5$\;\begin{array}{c} f\\ a,b \end{array}$	38 ± 9$\;\begin{array}{c} c\\ a,b,c \end{array}$	86 ± 3$\;\begin{array}{c} g\\ d \end{array}$	67 ± 2$\;\begin{array}{c} b\\ a,b,c,d \end{array}$	32 ± 4$\;\begin{array}{c} e\\ a \end{array}$	80 ± 13$\;\begin{array}{c} a,b\\ c,d \end{array}$	63 ± 10$\;\begin{array}{c} e\\ a,b,c,d \end{array}$	58 ± 2$\;\begin{array}{c} e\\ a,b,c,d \end{array}$
Lasiocarpine *N*-oxide	15 ± 3$\;\begin{array}{c} e,f\\ a,b \end{array}$	63 ± 6$\;\begin{array}{c} g\\ c,d \end{array}$	2947 ± 76$\;\begin{array}{c} g\\ e \end{array}$	45 ± 4$\;\begin{array}{c} e\\ b,c,d \end{array}$	10 ± 2$\;\begin{array}{c} c\\ a \end{array}$	25 ± 5$\;\begin{array}{c} b\\ a,b \end{array}$	30 ± 8$\;\begin{array}{c} e\\ a,b \end{array}$	13 ± 1$\;\begin{array}{c} a\\ a,b \end{array}$	27 ± 2$\;\begin{array}{c} d\\ a,b \end{array}$	31 ± 1$\;\begin{array}{c} a,b\\ a,b \end{array}$	27 ± 1$\;\begin{array}{c} d\\ a,b \end{array}$	72 ± 4$\;\begin{array}{c} f\\ d \end{array}$
Total	376 ± 13	4239 ± 17	6375 ± 168	730 ± 15	334 ± 8	340 ± 31	722 ± 17	1041 ± 16	450 ± 14	2128 ± 236	1199 ± 19	928 ± 16

n.d. = not detected; <MQL: below the limit of quantification of the method. In the sample identification code, the first letter indicates: W for wild farming, O for organic farming and C for conventional farming. Samples ending with an A or B indicate same trademark samples with different lot number. Different superscript letters in the same column indicate significant differences (*p* < 0.05) among PAs/PANOs in each sample. Different subscript letters in the same row indicate significant differences (*p* < 0.05) among samples.

## Supplementary Materials

The following are available online at https://www.mdpi.com/2304-8158/9/9/1319/s1, Table S1: oregano food alerts reported on the Food and Feed Safety Alerts (RASFF) portal related to the presence of pyrrolizidine alkaloids (PAs) and their oxidized forms (pyrrolizidine alkaloids N-oxides, PANOS) during 2019–2020; Table S2: information of the oregano samples analyzed; Table S3: solvent-based standard calibration curves; Figure S1: extracted ion chromatograms and mass spectra (MS2) of: intermedine (1), lycopsamine (2), europine (3), europine N-oxide (4) intermedine N-oxide (5), lycopsamine N-oxide (6), retrorsine (7), retrorsine N-oxide (8), seneciphylline (9), heliotrine (10), seneciphylline N-oxide (11), heliotrine N-oxide (12), senecivernine (13), senecionine (14), senecivernine *N*-oxide (15), senecionine *N*-oxide (16), echimidine (17), echimidine *N*-oxide (18), senkirkin (19), lasiocarpine (20) and lasiocarpine *N*-oxide (21) in a standard solution containing 100 µg/L of each analyte.
