# Supplementary material for: A Miniaturized QuEChERS Method Combined with Ultrahigh Liquid Chromatography Coupled to Tandem Mass Spectrometry for the Analysis of Pyrrolizidine Alkaloids in Oregano Samples
Source: Foods. 2020 Sep 18;9(9):1319. doi: 10.3390/foods9091319 (PMC7554850; doi:10.3390/foods9091319)
Supplement: Supplementary file 1 [file foods-09-01319-s001.pdf]

Supplementary material

Table S1. Oregano food alerts reported on the Food and Feed Safety Alerts (RASFF) portal related to the presence of pyrrolizidine alkaloids (PAs) and their oxidized forms (pyrrolizidine alkaloids *N*-oxides, PANOS) during 2019-2020. Data obtained online from: https://webgate.ec.europa.eu/rasff-window/portal/?event=SearchForm&cleanSearch=1 (accessed on 28 May 2020)

Search criteria | **Hazard category** natural toxins (other) | **Risk decision** serious

product category	date	reference	product type	notification type	notification basis	notified by	countries concerned	subject	action taken	distribution status	risk decision
herbs and spices	28/01/2019	2019.0305	food	alert	company's own check	Germany	Austria (O), Belgium (D), Czech Republic (D), France (D), Germany (D/O), INFOSAN, Netherlands (D), Slovakia (D), Turkey (O), Ukraine (D)	pyrrolizidine alkaloids (35447; 42724 µg/kg - ppb) in oregano from Austria and Turkey, processed in Germany	withdrawal from the market	distribution to other member countries	serious
herbs and spices	12/02/2019	2019.0504	food	alert	official control on the market	Germany	Austria (D), Commission Services, France (D), Germany (D), INFOSAN, Italy (D), Turkey (O)	pyrrolizidine alkaloids (6660 µg/kg - ppb) in oregano from Turkey	withdrawal from the market	distribution to other member countries	serious
herbs and spices	25/03/2019	2019.1121	food	information for attention	company's own check	Germany	Germany (D), INFOSAN, Turkey (O)	pyrrolizidine alkaloids (16896 µg/kg - ppb) in dried oregano from Turkey	detained by operator	distribution restricted to notifying country	serious
herbs and spices	09/05/2019	2019.1736	food	alert	official control on the market	Austria	Austria (D), France (O), Germany (D)	pyrrolizidine alkaloids (14871 µg/kg - ppb) in oregano from France	withdrawal from the market	distribution to other member countries	serious
herbs and spices	09/05/2019	2019.1739	food	alert	official control on the market	Austria	Austria (D/O), Germany (D)	pyrrolizidine alkaloids (14366 µg/kg - ppb) in oregano from Austria	detained by operator	distribution to other member countries	serious
herbs and spices	09/05/2019	2019.1741	food	alert	official control on the market	Austria	Austria (D/O), Germany (D)	pyrrolizidine alkaloids (21011 µg/kg - ppb) in rubbed oregano from Austria	detained by operator	distribution to other member countries	serious
herbs and spices	31/05/2019	2019.1997	food	information for attention	official control on the market	Germany	Germany (D), INFOSAN, Turkey (O)	pyrrolizidine alkaloids (9870 µg/kg - ppb) in oregano from Turkey	withdrawal from the market	distribution restricted to notifying country	serious
herbs and spices	03/06/2019	2019.2012	food	information for attention	official control on the market	Germany	Germany (D), INFOSAN, Turkey (O)	pyrrolizidine alkaloids (13100 µg/kg - ppb) in rubbed oregano from Turkey	withdrawal from the market	distribution restricted to notifying country	serious
herbs and spices	07/06/2019	2019.2099	food	alert	official control on the market	Germany	Austria (O), France (D), Germany (D), Italy (D)	pyrrolizidine alkaloids (11600 µg/kg - ppb) in oregano from Austria	withdrawal from recipient(s)	distribution to other member countries	serious
herbs and spices	20/06/2019	2019.2235	food	alert	official control on the market	Germany	Austria (D), Belgium (D), Cyprus, France (D), Germany (D), INFOSAN, Netherlands (D), Turkey (O)	pyrrolizidine alkaloids (11700 µg/kg - ppb) in oregano from Turkey	withdrawal from recipient(s)	distribution to other member countries	serious
herbs and spices	08/07/2019	2019.2458	food	information for attention	official control on the market	Germany	Germany (D), INFOSAN, Turkey (O)	pyrrolizidine alkaloids (10526 µg/kg - ppb) in oregano from Turkey	detained by operator	distribution restricted to notifying country	serious
herbs and spices	10/07/2019	2019.2504	food	alert	official control on the market	Austria	Austria (D), Germany (D), INFOSAN, Turkey (O)	pyrrolizidine alkaloids (15852 µg/kg - ppb) in oregano from Turkey	withdrawal from the market	distribution to other member countries	serious
herbs and spices	19/07/2019	2019.2635	food	information for attention	official control on the market	Germany	Germany (D), INFOSAN, Turkey (O)	pyrrolizidine alkaloids (13641 µg/kg - ppb) in oregano from Turkey	recall from consumers	distribution restricted to notifying country	serious
herbs and spices	30/07/2019	2019.2763	food	alert	official control on the market	Germany	Belgium (D), France (D), Germany (D), INFOSAN, Malta (D), Netherlands (D), Turkey (O), unknown origin	pyrrolizidine alkaloids (18496 µg/kg - ppb) in oregano from Turkey	recall from consumers	distribution to other member countries	serious
herbs and spices	07/08/2019	2019.2881	food	information for attention	official control on the market	Germany	Germany, INFOSAN, Turkey (O)	pyrrolizidine alkaloids (7590 µg/kg - ppb) in oregano from Turkey	return to consignor	product not (yet) placed on the market	serious
herbs and spices	26/09/2019	2019.3393	food	alert	company's own check	Germany	Austria (D), Belgium (D), Commission Services, Denmark (D), Germany, INFOSAN, Netherlands (D), Norway (D), Sweden (D), Turkey (O)	pyrrolizidine alkaloids (22167.4 µg/kg - ppb) in oregano from Turkey	informing recipient(s)	distribution to other member countries	serious
herbs and spices	29/01/2020	2020.0453	food	alert	official control on the market	Germany	Belgium (D), Denmark (D), France (D), Germany (D/O), Hungary (D), Luxembourg (D), Netherlands (D), Poland (D), Portugal (D), Slovenia (D), Spain (D), United Kingdom (D)	pyrrolizidine alkaloids (133870 µg/kg - ppb) in rubbed oregano from Germany	no stock left	distribution to other member countries	serious
herbs and spices	04/02/2020	2020.0568	food	alert	company's own check	Germany	Austria (D), Germany (D), INFOSAN, Turkey (O)	pyrrolizidine alkaloids (8836 µg/kg - ppb) in rubbed oregano from Turkey	withdrawal from the market	distribution to other member countries	serious
herbs and spices	05/02/2020	2020.0581	food	alert	official control on the market	Germany	France (D), Germany (D), INFOSAN, Turkey (O)	pyrrolizidine alkaloids (16962 µg/kg - ppb) in oregano from Turkey	withdrawal from the market	distribution to other member countries	serious

Table S2. Information of the oregano samples analyzed.

Oregano Sample	Product origin	Raw material origin	Description
C-S1	Spain	Unknown	Milled oregano leaf
C-S2	Italy	Unknown	Lyophilized oregano
C-S3	Spain	Spain	Milled oregano leaf
C-S4	Spain	Peru	Milled oregano leaf
C-S5	Spain	Peru	Milled oregano leaf
C-S6	Spain	Unknown	Milled oregano leaf
C-S7	Spain	Unknown	Milled oregano leaf
C-S8	Spain	Spain	Milled oregano leaf
C-S9	Spain	Spain	Milled oregano leaf
C-S10	Spain	Unknown	Milled oregano leaf
C-S11	Spain	Unknown	Milled oregano leaf
C-S12	Spain	Unknown	Milled oregano leaf
C-S13	France	Turkey	Milled oregano leaf
C-S14-A	Spain	France	Milled oregano leaf
C-S14-B	Spain	France	Milled oregano leaf
C-S15-A	Germany	Turkey	Milled oregano leaf
C-S15-B	Germany	Turkey	Milled oregano leaf
C-S16-A	Spain	Spain	Milled oregano leaf
C-S16-B	Spain	Spain	Milled oregano leaf
O-S17	Spain	Unknown	Milled oregano leaf from organic farming
O-S18	Spain	Spain	Milled oregano leaf from organic farming
O-S19	Spain	Unknown	Milled oregano leaf from organic farming
W-S20	Spain	Spain	Wild oregano on branch

Table S3. Solvent-based standard and matrix-matched calibration curves (expressed at μg/L)

Analyte	Solvent-based standard calibration, R^2	Matrix-matched calibration, R^2
Intermedine	y = 27944 x – 53949<br>0.999	y = 6513 x + 5771<br>0.999
Europine	y = 61607 x – 49511<br>0.999	y = 14342 x + 58782<br>0.999
Lycopsamine	y = 27348 x - 25302<br>0.999	y = 4990 x + 8916<br>0.999
Europine *N*-oxide	y = 138491 x – 357700<br>0.998	y = 21811 x + 43628<br>0.999
Intermedine *N*-oxide	y = 40240 x + 144383<br>0.999	y = 6754 x + 75308<br>0.999
Lycopsamine *N*-oxide	y = 48020 x – 282150<br>0.999	y = 6454x + 29990<br>0.999
Retrorsine	y = 26655 x + 2816<br>0.999	y = 3493 x + 12358<br>0.999
Retrorsine *N*-oxide	y = 9764 x + 40585<br>0.999	y = 1739 x + 13424<br>0.999
Seneciphylline	y = 61799 x – 12213<br>0.998	y = 7181 x + 25457<br>0.999
Heliotrine	y = 88524 x + 33860<br>0.998	y = 15268 x + 9413<br>0.999
Seneciphylline *N*-oxide	y = 27270 x + 27109<br>0.998	y = 9139 x - 29835<br>0.999
Heliotrine *N*-oxide	y = 68052 x – 183194<br>0.999	y = 2189 x + 13923<br>0.999
Senecivernine	y = 100194 x – 46983<br>0.998	y = 24972 x + 480780<br>0.999
Senecionine	y = 143096 x + 2237<br>0.998	y = 25056 x + 339046<br>0.999
Senecivernine *N*-oxide	y = 18958 x + 42888<br>0.999	y = 7319 x + 4421<br>0.999
Senecionine *N*-oxide	y = 13974 x + 34238<br>0.999	y = 3865 x + 146398<br>0.999
Echimidine	y = 88932 x – 223173<br>0.998	y = 15054 x + 167863<br>0.999
Echimidine *N*-oxide	y = 2911 x + 64931<br>0.999	y = 3217 x - 48988<br>0.999
Senkirkin	y = 54118 x + 412125<br>0.998	y = 4247 x + 17984<br>0.999
Lasiocarpine	y = 5028 x – 24107<br>0.998	y = 902 x - 4928<br>0.999
Lasiocarpine *N*-oxide	y = 150969 x + 113710<br>0.999	y = 20006 x + 2254<br>0.999

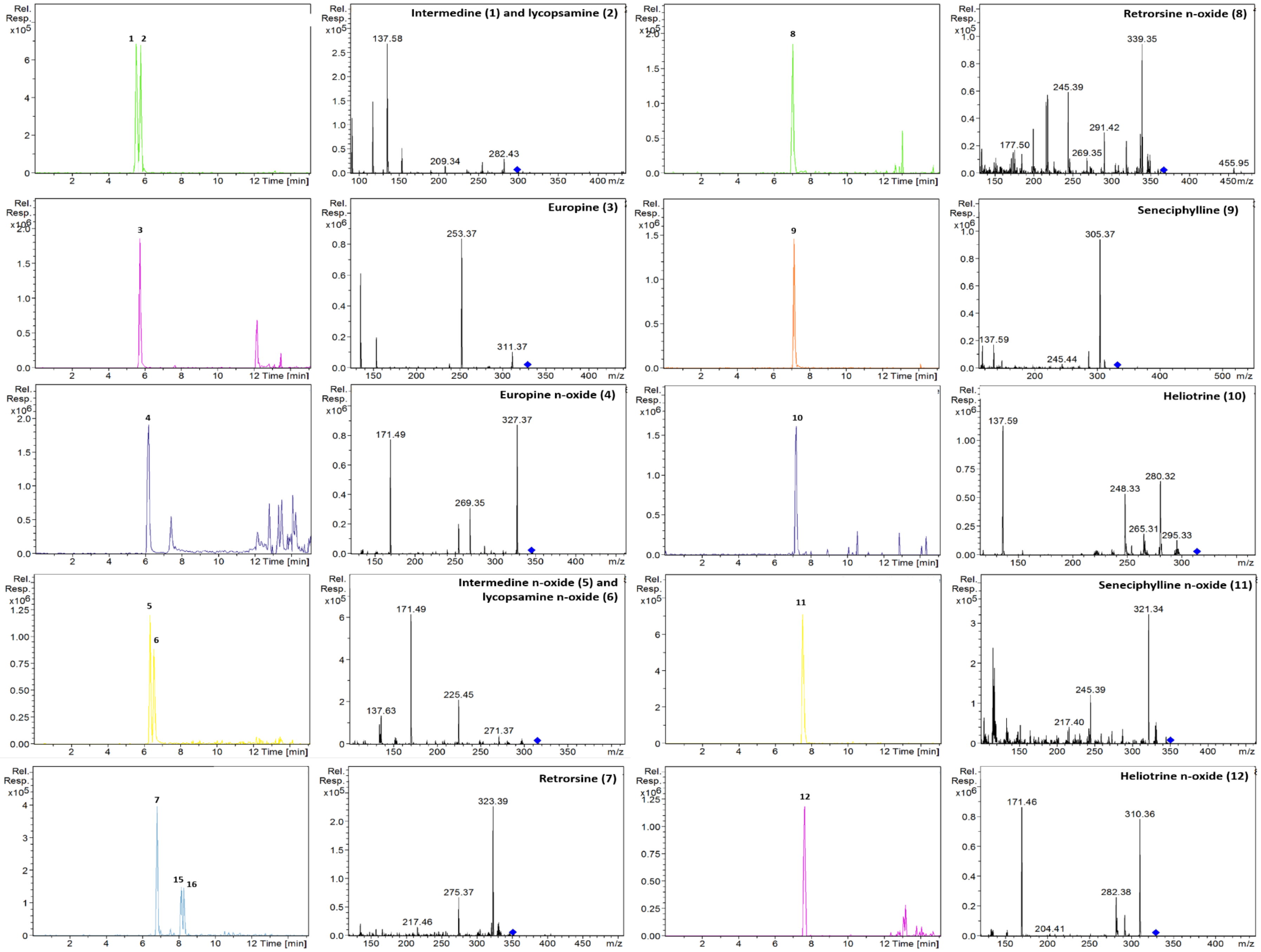

Intermedine (1) and lycopsamine (2)
137.58
209.34
282.43
Retrorsine n-oxide (8)
339.35
245.39
291.42
177.50
269.35
455.95
Europine (3)
253.37
311.37
Seneciphylline (9)
305.37
137.59
245.44
Europine n-oxide (4)
171.49
327.37
269.35
Heliotrine (10)
137.59
280.32
248.33
265.31
295.33
Intermedine n-oxide (5) and lycopsamine n-oxide (6)
171.49
137.63
225.45
271.37
Seneciphylline n-oxide (11)
321.34
245.39
217.40
Retrorsine (7)
323.39
275.37
217.46
Heliotrine n-oxide (12)
171.46
310.36
282.38
204.41
1
2
3
4
5
6
7
8
9
10
11
12
15
16
Rel. Resp.
Time [min]
m/z

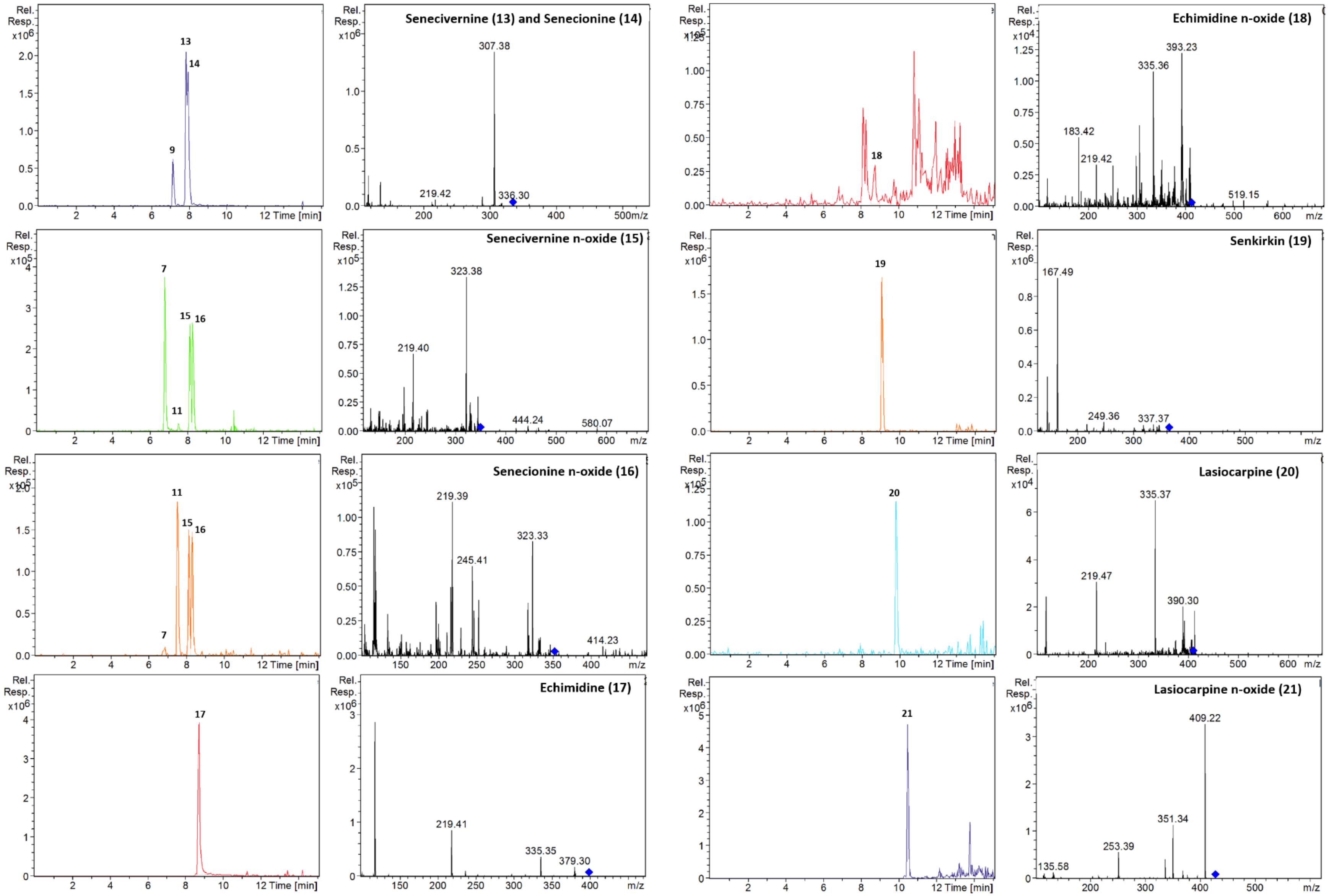

Figure S1. Extracted ion chromatograms and mass spectra (MS²) of: intermedine (1), lycopsamine (2), europine (3), europine *N*-oxide (4) intermedine *N*-oxide (5), lycopsamine *N*-oxide (6), retrorsine (7), retrorsine *N*-oxide (8), seneciphylline (9), heliotrine (10), seneciphylline *N*-oxide (11), heliotrine *N*-oxide (12), senecivernine (13), senecionine (14), senecivernine *N*-oxide (15), senecionine *N*-oxide (16), echimidine (17), echimidine *N*-oxide (18), senkirkin (19), lasiocarpine (20) and lasiocarpine *N*-oxide (21) in a standard solution containing 100 µg/L of each analyte.
